# Blood-Brain Barrier Dysfunction Amplifies the Development of Neuroinflammation: Understanding of Cellular Events in Brain Microvascular Endothelial Cells for Prevention and Treatment of BBB Dysfunction

**DOI:** 10.3389/fncel.2021.661838

**Published:** 2021-09-13

**Authors:** Fuyuko Takata, Shinsuke Nakagawa, Junichi Matsumoto, Shinya Dohgu

**Affiliations:** Department of Pharmaceutical Care and Health Sciences, Faculty of Pharmaceutical Sciences, Fukuoka University, Fukuoka, Japan

**Keywords:** blood-brain barrier, pericyte, astrocyte, microglia, oligodendrocyte, tight junction, neuroinflammation, cytokine

## Abstract

Neuroinflammation is involved in the onset or progression of various neurodegenerative diseases. Initiation of neuroinflammation is triggered by endogenous substances (damage-associated molecular patterns) and/or exogenous pathogens. Activation of glial cells (microglia and astrocytes) is widely recognized as a hallmark of neuroinflammation and triggers the release of proinflammatory cytokines, leading to neurotoxicity and neuronal dysfunction. Another feature associated with neuroinflammatory diseases is impairment of the blood-brain barrier (BBB). The BBB, which is composed of brain endothelial cells connected by tight junctions, maintains brain homeostasis and protects neurons. Impairment of this barrier allows trafficking of immune cells or plasma proteins into the brain parenchyma and subsequent inflammatory processes in the brain. Besides neurons, activated glial cells also affect BBB integrity. Therefore, BBB dysfunction can amplify neuroinflammation and act as a key process in the development of neuroinflammation. BBB integrity is determined by the integration of multiple signaling pathways within brain endothelial cells through intercellular communication between brain endothelial cells and brain perivascular cells (pericytes, astrocytes, microglia, and oligodendrocytes). For prevention of BBB disruption, both cellular components, such as signaling molecules in brain endothelial cells, and non-cellular components, such as inflammatory mediators released by perivascular cells, should be considered. Thus, understanding of intracellular signaling pathways that disrupt the BBB can provide novel treatments for neurological diseases associated with neuroinflammation. In this review, we discuss current knowledge regarding the underlying mechanisms involved in BBB impairment by inflammatory mediators released by perivascular cells.

## Introduction

Neuroinflammation is widely observed in neurodegenerative diseases including Alzheimer’s disease (AD), Parkinson’s disease (PD), multiple sclerosis (MS), and amyotrophic lateral sclerosis (ALS). Although inflammatory responses in the central nervous system (CNS) are essentially beneficial for repair of damaged brain tissue, inappropriate inflammatory responses can lead to neuronal dysfunction. CNS homeostasis depends on the balance of innate immunity. Innate immunity is the first line of defense against exogenous pathogens. Pathogen recognition receptors (PRRs) expressed on glial cells and neurons become activated by binding to their ligands, pathogen-associated molecular patterns (PAMPs). PRRs also recognize endogenous substances like damage-associated molecular patterns (DAMPs), leading to sterile inflammation of the brain. Binding of DAMPs to PRRs triggers the activation of glial cells (astrocytes and microglia), which is recognized as a hallmark of neuroinflammation and induces the release of inflammatory mediators and cytotoxic factors that contribute to neurodegenerative pathology (Chitnis and Weiner, 2017; Liddelow et al., 2017). Activated glial cells act as a major source of inflammatory mediators and affect the brain microvascular cells, that compose the blood-brain barrier (BBB), as well as neurons. Glial cell-derived inflammatory mediators including cytokines, chemokines, reactive oxygen species (ROS), and lipid mediators influence BBB integrity.

### Neuroinflammation and BBB Dysfunction in Various Diseases

Accumulating evidence has indicated that disruption of the BBB is a common feature of neuroinflammation-mediated neurodegeneration. BBB disruption is observed in patients with neurodegenerative diseases including AD (Bowman et al., 2007), PD (Gray and Woulfe, 2015), ALS (Zlokovic, 2008), and MS (Tofts and Kermode, 1991; Waubant, 2006). The BBB is a highly sophisticated system that restricts the transport of certain plasma proteins and immune cells from the blood to the brain parenchyma. BBB disruption is considered to allow lymphocytes, macrophages, and plasma proteins to enter the brain parenchyma. Infiltration of peripheral immune cells was shown to induce microglial activation (Laurent et al., 2017) and neurodegeneration (Mayne et al., 2020). Various neuroimaging molecules or methods for detecting BBB dysfunction and neuroinflammation in neurodegenerative diseases have been well reviewed elsewhere (Sweeney et al., 2018; Werry et al., 2019).

#### Neuroinflammation and BBB Dysfunction in Traumatic Brain Injury

Traumatic brain injury (TBI) is classified as mild, moderate and severe TBI. BBB dysfunction is widely observed from mild to severe TBI (O’Keeffe et al., 2020). BBB disruption in mild TBI precedes neuroinflammation responses identified by microglia and astrocyte activation (Wu et al., 2021). BBB disruption occurs within hours after brain injury and can be sustained for years (Hay et al., 2015). Extravasation of plasma proteins including fibrinogen, IgG, and albumin has been observed in the brain of patients with TBI in the acute and chronic phase. TBI-induced neuroinflammation responses can be identified by elevated levels of inflammatory mediators, within hours post-injury (Corrigan et al., 2016; Lier et al., 2020). Microglia and astrocytes are activated by the extravasation of albumin to release cytokines/chemokines and matrix metalloproteinases (MMPs) which disrupt the BBB (Ralay Ranaivo et al., 2012).

#### Neuroinflammation and BBB Dysfunction in Stroke

In acute ischemic stroke, BBB breakdown occurs within several hours of ischemic onset. This allows plasma proteins to enter the brain parenchyma resulting in vasogenic edema. Early BBB breakdown is mainly due to oxidative stress including ROS. ROS modulate tight junction proteins and the cytoskeleton of brain endothelial cells. Subsequently, neuroinflammation processes induce further BBB breakdown over the following 72 h (da Fonseca et al., 2014). Ischemia-induced cell death, DAMPs, miRNA and oxidative stress activate microglia and astrocytes, leading to the production of inflammatory cytokines. Glial cell-derived matrix metalloproteinases (MMPs) contribute to BBB dysfunction through digesting tight junction proteins at this phase. Neuroinflammatory responses in stroke occur over days to weeks (Xing et al., 2012). BBB dysfunction is sustained during the chronic stages of stroke (Strbian et al., 2008).

Following these acute CNS injuries described above, cerebral edema as a result of BBB dysfunction develops through several phases, including ionic edema, vasogenic edema and hemorrhagic conversion (Stokum et al., 2016). Brain endothelial dysfunction contributes to these processes. In ionic edema which occurs with an intact BBB, ions (Na^+^ and Cl^–^) and water flux are increased though brain endothelial ion channels and transporters such as Na^+^/H^+^ exchanger, Na^+^/K^+^-ATPase, Na^+^/K^+^Cl^–^ co-transporter, Sur1-Trpm4, GLUT1 and SGLT1. Perivascular astrocytic aquaporin 4 also contributes to ionic edema formation (Haj-Yasein et al., 2011). Vasogenic edema is characterized by BBB breakdown and extravasation of plasma proteins. This subtype of cerebral edema is mediated by MMP-9 and several inflammatory mediators (Stokum et al., 2016). Intracerebral hemorrhage occurs due to a loss of structural integrity of brain microvessels and allows extravasation of all component of blood. Infiltrating erythrocytes and plasma proteins triggers glial activation and neuronal damage.

#### Neuroinflammation and BBB Dysfunction in Neurodegenerative Diseases

Multiple sclerosis is an autoimmune and neuroinflammatory disease leading to demyelination and neurodegeneration. BBB dysfunction is an early feature of MS pathogenesis (Alvarez et al., 2011) and triggers immune cell infiltration and plasma protein extravasation. This is supported by fibrinogen deposition in developing lesions (Vos et al., 2005). Infiltrated immune cells release various inflammatory mediators and activate astrocytes and microglia, which contribute to progressive MS (Correale et al., 2017). These events would exacerbate BBB dysfunction.

Amyotrophic lateral sclerosis is characterized by progressive motor neurodegeneration in the brain and spinal cord. Various mutant genes are involved in the inflammatory responses which contribute to the pathogenesis of ALS (Beers and Appel, 2019). Post mortem tissue analysis revealed that BBB dysfunction occurs in gray and white matter of patient with ALS (Garbuzova-Davis et al., 2012). ALS model mice showed that damage in neurovascular unit occurred prior to neurodegeneration (Miyazaki et al., 2011). Activation of microglia and astrocytes in living patients with ALS was detected by use of ^11^C-PK11195 PET imaging (Turner et al., 2004) and ^11^C-Deuterium-L-Deprenyl (DED) PET imaging (Johansson et al., 2007), respectively.

The main pathological hallmarks of PD are dopaminergic neuronal loss and deposition of Lewy bodies, which are mainly composed of aggregated α-synuclein. α-Synuclein induces neuroinflammation associated with PD development (Su et al., 2008). Extracellular α-synuclein activates microglia and astrocytes through Toll-like receptors to release inflammatory mediators (Lee et al., 2010; Fellner et al., 2013). Monomeric α-synuclein induces the release of inflammatory mediators from brain pericytes leading to rat brain endothelial barrier dysfunction (Dohgu et al., 2019). One previous study reported that fibrillar α-synuclein-induced dysfunction of the human brain endothelial barrier when co-cultured with neurons (Kuan et al., 2016). In addition, BBB leakage of fibrin and hemosiderin was observed in the striatum of PD patients (Gray and Woulfe, 2015). Subtle BBB disruption in PD patients is observed in the substantia nigra, white matter and posterior cortical regions (Al-Bachari et al., 2020).

Alzheimer’s disease is characterized by the increased production and deposition of misfolded protein amyloid β (Aβ) and tau, and extracellular deposits of Aβ induce neuroinflammation. Activated microglia and astrocytes, as well as the release of various inflammatory cytokines around Aβ plaques, have been observed in AD brains (da Fonseca et al., 2014). In addition, Aβ is reported to impair the integrity of BBB (Chan et al., 2018; Cuevas et al., 2019). According to the two-hit vascular hypothesis in AD (Nelson et al., 2016), BBB dysfunction leads to the infiltration of neurotoxic substances in brain parenchyma and triggers neuroinflammation. Deposition of harmful plasma proteins like fibrin has been observed in a wide range of neurodegenerative diseases (Petersen et al., 2018). Fibrinogen entering the CNS was found to promote microglial activation, leading to the release of inflammatory mediators that caused neuronal damage in AD mice (Merlini et al., 2019). Fibrinogen is not expressed in the brain, and therefore BBB dysfunction is necessary for plasma fibrinogen to cross the BBB and enter the brain parenchyma. Measurement of BBB integrity with dynamic contrast-enhanced magnetic resonance imaging revealed increased BBB permeability to a gadolinium-based contrast agent in the hippocampus in patients with mild cognitive impairment (Montagne et al., 2015). These findings suggest that a lesser extent of BBB dysfunction that allows penetration of small molecules, but not large molecules like fibrinogen, precedes the development of AD.

#### BBB Dysfunction Amplifies Neuroinflammation

**FIGURE 1:**
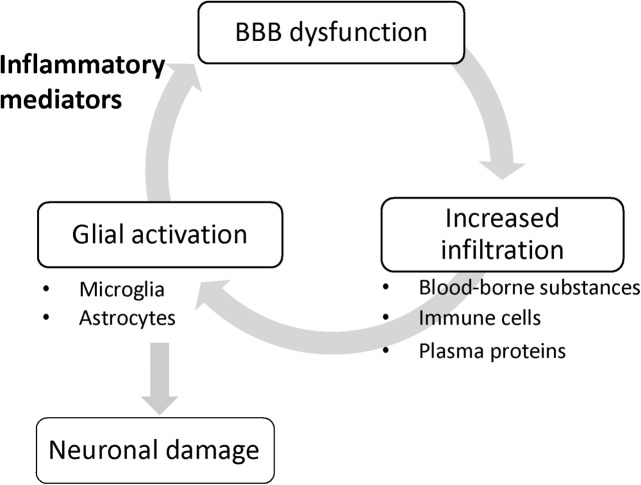
BBB dysfunction amplifies glial activation. Once glial cells are activated without any impairment of the brain endothelial barrier function, glial activation triggers BBB dysfunction through the release of inflammatory mediators, and in turn, the leaky BBB allows infiltration of blood-borne inducers of glial activation into the brain, resulting in aggravated glial activation.

It remains unclear whether the lesser extent of BBB dysfunction is a cause or a result of the neuroinflammation and neurodegeneration. Importantly, systemic administration of lipopolysaccharide (LPS), a gram-negative bacterial endotoxin, caused increased BBB permeability to sodium fluorescein in parallel with an increased number of activated microglia (Nishioku et al., 2009). Ju et al. (2018) reported that increased BBB permeability induced by mannitol led to microglial activation. At the least, these findings suggest that a leaky BBB contributes to microglial activation and has the potential for understanding how to reduce the exacerbation of sterile brain inflammation initiated by activation of innate immune responses in glial cells. Once glial cells are activated without any impairment of the brain endothelial barrier function, glial activation triggers BBB dysfunction through the release of inflammatory mediators, and in turn, the leaky BBB allows infiltration of blood-borne inducers of glial activation into the brain, resulting in aggravated brain inflammation and glial activation (Figure 1). Thus, BBB dysfunction positively amplifies the development of neuroinflammation, rather than being a pathological result of glial activation and neuroinflammation.

**FIGURE 2:**
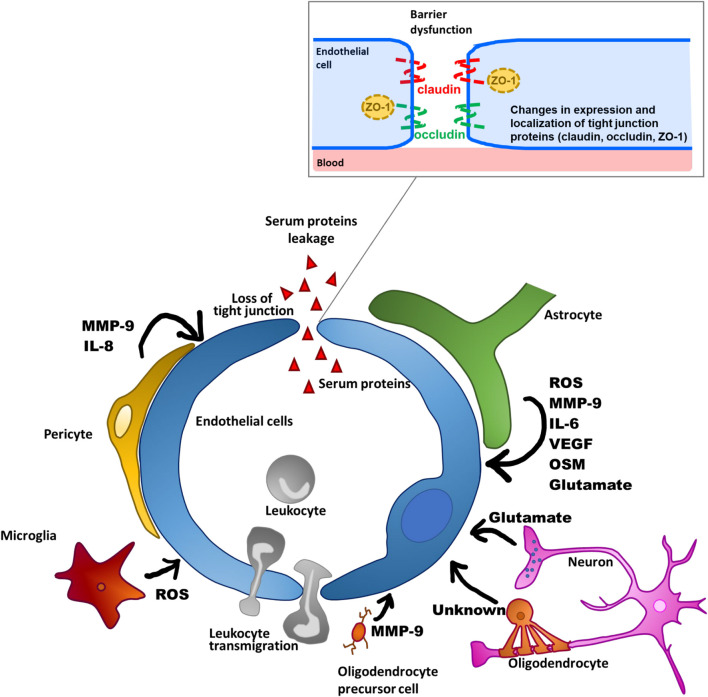
Schematic overview of BBB dysfunctions through factors produced by NVU-constituting cell types.

Given that the development of neurodegeneration is associated with neuroinflammation, pharmacological manipulation of BBB dysfunction would be a promising supportive treatment for neurodegenerative diseases. The barrier function of the BBB is mainly determined by tight junctions that seal paracellular gaps between brain microvascular endothelial cells (BMECs). Tight junctions are mainly composed of occludin and claudin-5 transmembrane proteins, and zonula occludens cytoplasmic scaffold proteins, such as ZO-1. The formation and maintenance of tight junctions is regulated by integration of multiple intercellular communications between BMECs and other brain cell types (pericytes, astrocytes, microglia, oligodendrocytes [OLs], and neurons) through various soluble factors (Luissint et al., 2012; Sweeney et al., 2019). However, these cells surrounding BMECs also participate in the process of BBB dysfunction through the release of various substances that affect BBB integrity, as described below and presented in Figure 2. In this review, we propose two components as therapeutic targets for BBB dysfunction: the non-cellular component, including inflammatory mediators derived from the surrounding cells, and the cellular component, referring to intracellular signaling pathways that lead to disruption of brain endothelial barrier integrity. In particular, we will focus on understanding of the intracellular signaling pathways that lead to BBB dysfunction for the purpose of pharmacologically targeting BMECs. Specifically, we will outline how the brain endothelial barrier is disrupted by all other brain parenchymal cell types, and then discuss inflammatory mediators and related intracellular signaling pathways that lead to BBB dysfunction, mainly with reference to *in vitro* studies, to clarify the cellular events in BMECs.

## Communications Between BMECs and Other Cell Types in Neurovascular Units Leading to BBB Dysfunction

### Pericytes

Pericytes surround brain microvessels and communicate with BMECs to stabilize functional microvessels through physical contacts such as *N*-cadherin-dependent adherens junctions and connexin 43-dependent gap junctions, as well as paracrine signaling *via* soluble factors and their receptors (Caruso et al., 2009; Navarro et al., 2016; Perrot et al., 2020). Previous studies of pericyte-deficient transgenic mice revealed BBB hyperpermeability to neurotoxic serum proteins like fibrin, thrombin, and plasmin, indicating that the presence of brain pericytes in brain microvessels is integral to BBB integrity and normal neuronal function (Bell et al., 2010). Furthermore, in the cortex of pericyte-deficient mice, neuroinflammatory molecules like tumor necrosis factor-α (TNF-α), monocyte-chemoattractant protein (MCP)-1, and intercellular adhesion molecule 1 (ICAM-1) were elevated, and increased Iba1 expression, an index of microglial activation, was observed with age. These findings suggest that the brain pericyte deficit led to impaired communication with BMECs and evoked neuronal inflammation through BBB impairment. Indeed, sepsis model mice showed microglial activation and brain pericyte detachment from the basal lamina following elevation of BBB permeability (Nishioku et al., 2009). Thus, the insufficient pericyte coverage of microvessels observed in CNS disorders like ischemia and TBI (Fernández-Klett et al., 2013; Zehendner et al., 2015) may be implicated in the development of BBB dysfunction and brain inflammation.

Pericytes in the brain microvessels are also likely to contribute to induction of BBB dysfunction under pathological conditions, even though brain pericyte detachment from the basal lamina was not observed. Brain pericytes express several receptors for endogenous cytokines, pathogens, and pathogenic molecules like TNF-α, interleukin (IL)-1β, IL-8, LPS, and α-synuclein, and are implicated in the production of inflammation-related molecules (Matsumoto et al., 2014; Navarro et al., 2016; Dohgu et al., 2019). Interestingly, brain pericytes were much more responsive than other cell types constituting the BBB, such as BMECs and astrocytes, to TNF-α and thrombin stimulation in producing MMP-9 (Takata et al., 2011; Machida et al., 2015). Under pathological conditions, production of MMP-9 in the brain leads to BBB impairment through rearrangement and degradation of tight junction-associated proteins (Bauer et al., 2010; Zhang et al., 2012). Obesity-associated diabetes mice had elevated thrombin levels in their brain and BBB dysfunction, suggesting that BBB dysfunction could be attributed to the release of MMP-9 by thrombin-reactive pericytes (Machida et al., 2017b). Indeed, *in vitro* studies demonstrated that thrombin-induced MMP-9 production by brain pericytes led to increased BBB permeability through morphological disorganization of ZO-1 and decreased expression of ZO-1 and occludin in BMECs (Machida et al., 2017a, b). Based on these findings, the possibility that pericytes localized in microvessels can act as inducers and amplifiers of BBB dysfunction through the release of molecules that lead to increased BBB permeability in BMECs under pathological conditions should be considered.

Among the CNS-constituting cell types, including BMECs, astrocytes, and microglia, pericytes are the most sensitive to TNF-α and have a unique cytokine and chemokine release profile, characterized by substantial release of IL-6 and macrophage inflammatory protein-1α (Matsumoto et al., 2014). Furthermore, in a pericyte/microglia co-culture system, TNF-α treatment induced increased expression of inducible nitric oxide synthase (iNOS) and IL-1β in the microglia as an index of microglial activation, whereas the induction was not observed in microglia monoculture in the absence of pericytes. TNF-α-sensitive pericytes evoked microglial activation accompanied by increased iNOS through cooperation between the IκB-nuclear factor kappa-light-chain enhancer of activated B cells (NF-κB) and Janus-activated kinase (JAK)-signal transducer and activator of transcription 3 (STAT3) pathways (Matsumoto et al., 2018). In TBI mice, an injury-induced pericyte response characterized by rapidly increased platelet-derived growth factor receptor β (PDGFRβ) expression was observed, followed by activation of microglia (Sakai et al., 2021). Inhibition of PDGFRβ signaling in reactive pericytes in TBI mice suppressed the microglial activation, raising the possibility that the response of pericytes to pathological insults mediates the process of brain inflammation including microglial activation (Sakai et al., 2021). Taken together, accumulating evidence suggests that brain pericytes play a key role as mediators of neuroinflammation.

As well as contributing to propagation of inflammatory responses in the brain *via* the release of inflammatory mediators and activation of microglia, brain pericytes are essential players in the regulation of leukocyte diapedesis in brain tissue (Rudziak et al., 2019). Brain pericytes stimulated by inflammatory mediators like TNF-α, IL-1β, and LPS produce IL-8 and MMP-9 (Pieper et al., 2013). In a pericyte/endothelial cell coculture system, these inflammatory mediators increased neutrophil transmigration, while the addition of neutralizing antibodies against IL-8 attenuated the enhanced neutrophil transmigration. Meanwhile, inhibition of MMP-9 derived from brain pericytes treated with these mediators enhanced adhesion of neutrophils to pericytes, suggesting the possibility that MMP-9 derived from pericytes releases neutrophils attached to pericytes in the brain parenchyma. Collectively, inflammatory reactive pericytes may allow leukocytes to breach the BBB through the released IL-8 and MMP-9, leading to penetration of leukocytes into the brain and the subsequent development of neuroinflammation.

### Astrocytes

Astrocytes exist around brain microvessels and are one of the important cellular constituents of the BBB together with brain pericytes. The terminal processes of astrocytes, known as endfeet, express potassium channels and aquaporin-4, which support BBB functions by controlling the ion and water balance (Michinaga and Koyama, 2019). In the healthy adult brain, it is commonly assumed that astrocytes play a role in maintaining BBB integrity, because the integrity in an *in vitro* BBB model involving co-culture of endothelial cells and astrocytes was greater than that in monoculture of endothelial cells (Nakagawa et al., 2009). Studies demonstrated that astrocyte-derived soluble factors, such as glial-derived neurotrophic factor, fibroblast growth factor, and angiopoietin 1, were partly responsible for modulating BBB functions (Igarashi et al., 1999; Lee et al., 2003; Murakami et al., 2008; Lecuyer et al., 2016).

Under pathological conditions, including ischemic injury, reactive astrocytes were shown to be enhanced (Liu M. et al., 2020) and to secrete inflammatory factors like vascular endothelial growth factor A (VEGF-A), MMPs and MCP-1 (Shan et al., 2019), which can directly or indirectly aggravate BBB disruption. Mice subjected to middle cerebral artery occlusion exhibited increased VEGF-A and MMP-9 expression in astrocytes. Astrocytes cultured under oxygen-glucose deprivation plus reoxygenation conditions produced VEGF-A and MMP-9 *via* the JAK2/STAT3 signaling pathway. In both *in vivo* and *in vitro* brain ischemic models, reductions in VEGF-A and MMP-9 in astrocytes were shown to ameliorate the reduced BBB integrity induced by ischemic stress (Shan et al., 2019). Taken together, these findings directly implicate the astrocyte response to ischemic injury in BBB dysfunction through the production of VEGF-A and MMP-9. Furthermore, cytokines released by reactive astrocytes induce tight junction reorganization in the BBB. IL-6 derived from astrocytes elevated BBB permeability and induced the release of chemokines (Takeshita et al., 2017). Another cytokine in the IL-6 family, oncostatin M (OSM), was expressed in activated astrocytes from MS patients (Ensoli et al., 2002). Recently, OSM was also shown to increase BBB permeability and decrease expression of claudin-5 (Takata et al., 2008, 2018). Therefore, cytokines belonging to the IL-6 family are secreted by astrocytes and mediate BBB impairment during neuroinflammation.

A hallmark of cerebral inflammatory disorders such as MS and encephalopathy associated with virus infection is the appearance of immune cells derived from circulating blood in the brain, together with a loss of BBB integrity (Jaureguiberry-Bravo et al., 2016). Stimulation with TNF-α, IFNγ, and IL-1β enabled astrocytes to release chemoattractants including MCP-1 (Weiss et al., 1998). An *in vitro* study involving co-cultures of endothelial cells and astrocytes examined whether astrocytes can directly influence the transmigration of peripheral blood mononuclear cells (PBMCs) across the endothelial layer. Astrocyte-derived MCP-1 mediated greater monocyte migration across these co-cultures (Weiss et al., 1998). Conversely, in an *in vitro* co-culture model, the presence of astrocytes reduced Aβ-induced PBMC transmigration across endothelial layers as well as Aβ-induced ICAM-1 expression (Spampinato et al., 2019). These findings raise the possibility that astrocyte-derived factors play dual roles in mediating the transmigration of PBMCs across the BBB. Overall, astrocytes participate in both BBB impairment after pathological insults and the development of neuroinflammation.

### Microglia

Microglia, as resident immune cells in the brain, play important roles in primary immune responses to protect the CNS against insults such as infection, ischemia, injury, and disease. Resting microglia routinely survey their environments, and are ready to undergo rapid transformation to their activated state in response to inflammatory stimuli (Kettenmann et al., 2011). Activated microglia produce various inflammatory mediators, including cytokines, chemokines, and ROS, and subsequently induce neuroinflammation and neuronal damage (Subhramanyam et al., 2019). Emerging evidence has suggested that microglial activation and neuroinflammation are also associated with BBB dysfunction under pathological conditions (Zlokovic, 2008). Impairment of BBB integrity was shown to occur in parallel with microglial activation in LPS-induced septic encephalopathy model mice (Nishioku et al., 2009). Sumi et al. (2010) established a co-culture model using BMECs and rat primary microglia to investigate the direct interactions between activated microglia and BMECs. They found that LPS-activated microglia induced hyperpermeability to sodium fluorescein, a marker of the paracellular route between adjacent endothelial cells, and decreased the activity of P-glycoprotein in their co-culture system (Sumi et al., 2010; Matsumoto et al., 2012). The alterations by which LPS-activated microglia impaired the endothelial barrier functions were blocked by DPI, an inhibitor of nicotinamide adenine dinucleotide phosphate (NADPH) oxidase (Sumi et al., 2010; Matsumoto et al., 2012). They also found that blockade of TNF-α suppressed endothelial dysfunction by LPS-activated microglia (Nishioku et al., 2010). These findings indicate that microglial activation directly leads to brain endothelial barrier dysfunction. A recent study showed that LPS-activated microglia interacted with astrocytes and dramatically increased chemokine release in co-cultures of LPS-activated microglia and astrocytes. Furthermore, addition of LPS-activated microglia to the astrocyte layer in a BBB model (BMEC/pericyte/astrocyte triculture) decreased transendothelial electrical resistance (TEER) and increased sodium fluorescein permeability in the model (Shigemoto-Mogami et al., 2018). Thus, activated microglia directly and/or indirectly disrupt the integrity of the BBB through elevation of pro-inflammatory factors such as cytokines, chemokines and ROS.

### Oligodendrocyte Lineage Cells

Oligodendrocyte lineage cells including OLs and oligodendrocyte precursor cells (OPCs) are closely located to BMECs (Pham et al., 2012; Maki et al., 2015). Therefore, it is possible that oligodendrocyte lineage cells exert similar crosstalk with BMECs to pericytes and astrocytes, for the regulation of BBB functions. OPC-specific TGF-β-deficient mice exhibited loss of BBB functions and cerebral hemorrhage (Seo et al., 2014), suggesting that OPCs may be implicated in the upregulation of BBB functions through release of TGF-β. Indeed, in an *in vitro* study, OPC-conditioned medium decreased BBB permeability through BMECs and increased the expression of tight junction-associated proteins such as claudin-5, ZO-1, and occludin (Seo et al., 2014). Furthermore, OPCs exposed to PDGF-BB released from BMECs induced decreased BBB permeability in BMECs, indicating that BMECs can help OPCs to modulate BBB functions (Kimura et al., 2020). Thus, it is considered that the crosstalk between OPCs and BMECs *via* their secreted factors plays important roles in BBB formation and maintenance. In mice with cerebral ischemic injury, OPCs in the damaged white matter expressed increased MMP-9 at an early stage after injury and OPC-derived MMP-9 caused BBB impairment and neutrophil infiltration into the brain, leading to the development of brain dysfunctions, including demyelination (Seo et al., 2013). These findings suggest that stressed OPCs mediate BBB impairment in the injured white matter. Meanwhile, a recent study revealed that transplantation of healthy OPCs protected against BBB disruption in a mouse model of brain ischemia through activation of the Wnt7a/β-catenin pathway (Wang et al., 2020). These reports suggest that OPCs have a biphasic effect on BBB functions. Therefore, the endothelial BBB functions may be perturbed under pathological conditions in accordance with alterations in OPC functions in the CNS.

Besides OPCs, it was reported that the presence of OLs decreased BBB permeability *via* BMECs through unknown soluble factors (Kimura et al., 2020). Hence, OLs are expected to be implicated in the alterations to BBB functions under pathological conditions. However, there is no direct evidence linking altered interactions between BMECs and OLs with BBB impairment. Further experiments are required to understand the role of OLs in BBB pathology.

### Neurons

Brain microvessels has been shown to be positioned within 15 μm of every neuron. This proximity allows neuronal activity to regulate the brain endothelial function (Tsai et al., 2009). BMECs express receptors for neurotransmitters such as glutamate (Lu et al., 2019) and γ-aminobutyric acid (GABA) (Won et al., 2013) and can therefore sense neurotransmitters released from activated neurons. Increased BBB permeability was observed in neurological conditions associated with neuronal hyperexcitability during ischemia, trauma, and epileptic seizure (Tomkins et al., 2008; Friedman, 2011; Schoknecht et al., 2014). These findings raise the possibility that the occurrence of neural activity affects BBB functions. Exposure to glutamate, a major excitatory neurotransmitter, was shown to elicit reduced barrier function in cultured BMECs through activation of *N*-methyl-D-aspartic acid (NMDA) receptors following alterations in occludin expression and phosphorylation (András et al., 2007). Meanwhile, direct cortical imaging in rats revealed that cortical application of glutamate elevated BBB permeability to sodium fluorescein in a dose-dependent manner (Vazana et al., 2016). Taken together, these findings support the view that glutamate derived from activated neurons plays an important role in the mediation of BBB functions. Astrocytes also have the ability to release glutamate and drive this signaling by activating glutamate receptors in endothelial cells (Lu et al., 2019). Thus, further investigations are required to determine whether this neuronal activity is directly causative for the functional phenotype of the BBB under physiological and pathological conditions.

## Inflammatory Mediators Involved in BBB Dysfunction and Related Intracellular Signaling Pathways in BMECs

### IL-1β

**FIGURE 3:**
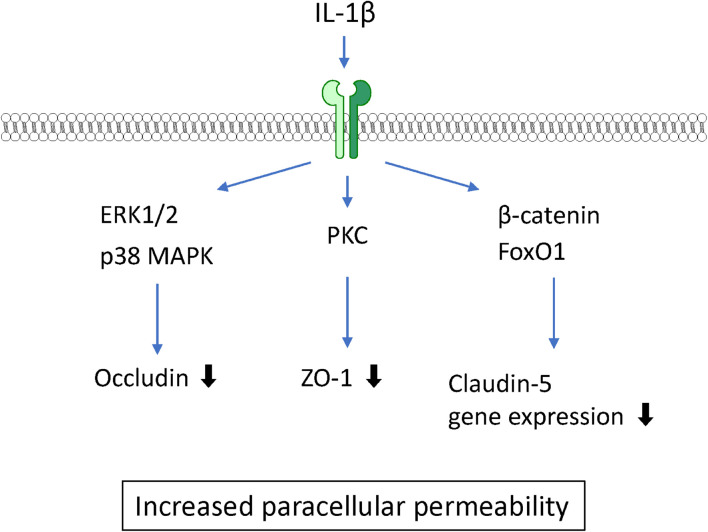
Intracellular signaling pathways induced by IL-1β in BMECs leading to BBB dysfunction.

IL-1β is widely recognized as a proinflammatory mediator associated with leakage of the BBB in CNS pathology in various animal studies. Intrastriatal injection of IL-1β induced a transient increase in BBB permeability (Blamire et al., 2000). IL-1β treatment of BMECs caused an increase in BBB permeability to sodium fluorescein and dextran (Beard et al., 2014; Ni et al., 2017) and a decrease in TEER (de Vries et al., 1996). These findings suggest that IL-1β acts directly on BMECs, resulting in increased BBB permeability. Although IL-1 type I and type II receptors are both expressed on BMECs, IL-1 type II receptors are involved in IL-1β transport across BMECs (Skinner et al., 2009). A 6-h exposure of BMECs to IL-1β did not affect the expression levels of claudin-5 and ZO-1, but did lead to IL-1β-induced brain endothelial barrier dysfunction associated with elevated phosphorylation of ZO-1 through activation of protein kinase C (PKC)θ (Rigor et al., 2012). A longer (24-h) exposure of BMECs to IL-1β decreased claudin-5 expression through transcriptional repression of β-catenin and FoxO1. IL-1β increased both β-catenin and FoxO1 nuclear translocation leading to decreased expression of claudin-5 mRNA. This suppression was dependent on non-muscle myosin light chain kinase (Beard et al., 2014). Furthermore, IL-1β decreased another tight junction-associated protein, occludin. The phosphorylation levels of ERK1/2 and p38 mitogen-activated protein kinase (MAPK) in BMECs were transiently elevated at 15–30 min after IL-1β treatment (Ni et al., 2017), suggesting that MAPK signaling is also involved in the IL-1β-induced BBB dysfunction (see Figure 3). Furthermore, IL-1β induced the expression of other inflammatory mediators including TNF-α, CXCL10, IL-8, IL-6, MCP-1, G-CSF, VEGF, and GM-CSF in BMECs (O’Carroll et al., 2015; Krasnow et al., 2017). Although it is not completely understood whether all of these BMEC-derived soluble factors induced by IL-1β disrupt the barrier integrity of BMECs, these factors may participate in the destructive effect of IL-1β on the BBB in an autocrine manner. Importantly, the ability of BMECs to secrete IL-1β in response to oxygen glucose deprivation and TNF-α should also be considered in IL-1β-induced BBB dysfunction (Matsumoto et al., 2014; Chen et al., 2017).

### TNF-α

TNF-α is well-known as a proinflammatory cytokine that induces BBB dysfunction. Accumulating evidence has indicated that various systemic and CNS inflammatory diseases induce BBB dysfunction through increased levels of TNF-α. An *in vitro* study using cultured BMECs showed that TNF-α directly interacted with TNF receptor I (p55) or TNF receptor II (p75) (Lucas et al., 1998) to generate increased BBB permeability (Deli et al., 1995b; Lopez-Ramirez et al., 2012; Ni et al., 2017) and decreased TEER (de Vries et al., 1996; Wong et al., 2004). Both TNF receptors act cooperatively to transport TNF-α across the BBB (Pan and Kastin, 2002). TNF-α decreased the expression of the tight junction-associated proteins ZO-1 (Rochfort and Cummins, 2015), claudin-5 (Aslam et al., 2012; Camire et al., 2015), and occludin (Ni et al., 2017). The underlying mechanisms for the TNF-α-induced reductions in tight junction-associated protein expression involve various intracellular signaling pathways. TNF-α reduced claudin-5 promoter activity and mRNA expression through NF-κB signaling (Aslam et al., 2012). Meanwhile, phosphatidylinositol-3 kinase (PI3K) inhibition with LY294002 attenuated the TNF-α-induced loss of claudin-5 expression in BMECs (Camire et al., 2015). These findings suggest that activation of NF-κB and PI3K induces BBB dysfunction mediated by TNF-α. BAY11-7058, an NF-κB inhibitor, inhibited the TNF-α-induced increase in BBB permeability (Coelho-Santos et al., 2015). Further studies involving cultures of BMECs are needed to determine whether pharmacological or genetic inhibition of PI3K activation in BMECs is linked to the attenuation of the increased BBB permeability induced by TNF-α. Although both ERK1/2 and p38 MAPK were phosphorylated by TNF-α, only p38 MAPK inhibition with the pharmacological inhibitor SB202190 attenuated the decreased expression of occludin induced by TNF-α without an inhibitory effect on the TNF-α-induced increase in paracellular permeability (Ni et al., 2017). TNF-α-induced activation of ERK1/2 likely mediates an increase in transcytosis (Miller et al., 2005) rather than an increase in paracellular permeability. The c-Jun *N*-terminal kinases (JNK) inhibitor SP600125 partially prevented the TNF-α-induced increase in paracellular permeability (Lopez-Ramirez et al., 2012). TNF-α also activated protein tyrosine kinase and PKC in BMECs (Hudson et al., 1996). Inhibition of PKC attenuated the TNF-α-induced endocytosis (Defazio et al., 2000), but not the paracellular hyperpermeability to fluorescein isothiocyanate (FITC)-dextran (Lopez-Ramirez et al., 2012) in BMECs. However, the PKCα/RhoA pathway mediated the TNF-α-induced F-actin rearrangement leading to decreased TEER (Peng et al., 2011).

**FIGURE 4:**
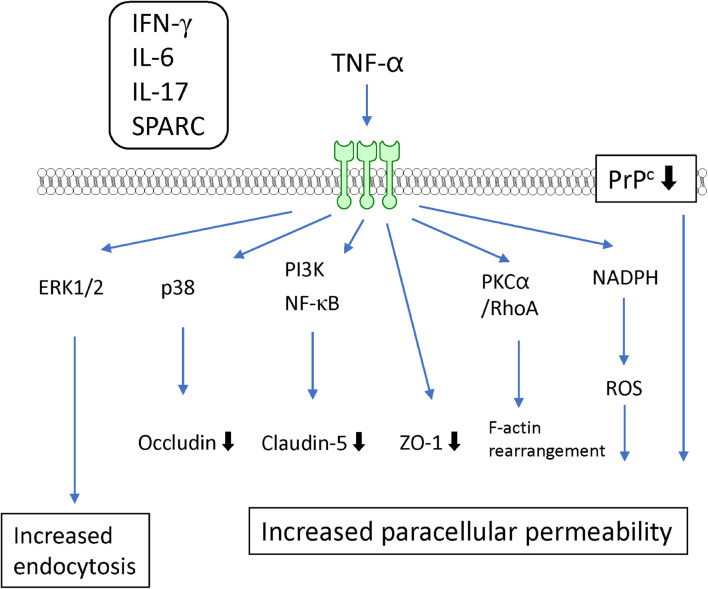
Intracellular signaling pathways induced by TNF-α in BMECs leading to BBB dysfunction. TNF-α induced release of IFN-γ, IL-6, IL-17 and SPARC by BMECs. They contribute to TNF-α-induced BBB dysfunction.

In addition to TNF-α-activated intracellular signaling pathways, other factors are involved in the TNF-α-induced BBB dysfunction. Treatment of BMECs with TNF-α induced the production of IL-6, leading to BBB disruption (Rochfort et al., 2016). Involvement of NADPH oxidase and ROS were also reported by the same research group (Rochfort et al., 2014; Rochfort and Cummins, 2015). Alkabie et al. (2016) reported that secreted protein acidic and rich in cysteine (SPARC), a cell-matrix-modulating protein, contributed to the TNF-α-induced BBB dysfunction. They found that TNF-α increased the expression of SPARC in BMECs. SPARC exhibited increased paracellular permeability in parallel with decreased expression of tight junction proteins (Alkabie et al., 2016). The TNF-α-induced decrease in expression of cellular prion protein (PrP^c^), a non-pathogenic cellular isoform constitutively expressed in BMECs, mediated the decreased expression of occludin and claudin-5 (Megra et al., 2018). Although the structural links between PrP^c^ and tight junction-associated proteins are unclear, such links are supported by evidence that PrP^c^ knockdown in BMECs altered the localization of tight junction proteins. TNF-α induced the production of various inflammatory mediators, including IL-6, IL-17, INF-γ, and MCP-1, in BMECs (Matsumoto et al., 2014). Indeed, combination with other inflammatory mediators (IL-6 and IL-17) potentiated the TNF-α-induced loss of BBB integrity through activation of NF-κB (Voirin et al., 2020). Co-treatment with IFN-γ and TNF-α induced hyperpermeability through activation of caspase-3 and caspase-9 (Lopez-Ramirez et al., 2012) (see Figure 4 and Table 1).

**TABLE 1:** Inflammatory mediators involved in BBB dysfunction and related cellular events in BMECs.

Mediators	Species	Cellular events in BMECs	References
IL-1β	Human	Permeability (FITC-Dextran) ↑ TEER ↓ Occludin expression ↓ Phosphorylation levels of ERK1/2 and p38 MAPK ↑	Ni et al., 2017
	Human	TEER ↓ Claudin-5 and ZO-1 expression → Phosphorylation of ZO-1 through activation of PKCθ↑	Rigor et al., 2012
	Porcine	TEER ↓ Transport of IL-1β through IL-1 type II receptor	Skinner et al., 2009
	Rat	TEER ↓	de Vries et al., 1996
	Mouse	Permeability (sodium fluorescein, TRITC-dextran) ↑ Permeability (albumin) → TEER ↓ Claudin-5 expression ↓ β-catenin and FoxO1 nuclear translocation ↑	Beard et al., 2014
TNF-α	Human	Permeability (FITC-dextran) ↑ TEER ↓ JNK signaling ↑ PKC signaling ↑ (not associated with TNF-α induced hyperpermeability)	Lopez-Ramirez et al., 2012
	Human	Permeability (FITC-dextran) ↑ TEER ↓ Occludin expression ↓ p38MAPK signaling ↑	Ni et al., 2017
	Human	TEER ↓	Wong et al., 2004
	Human	Permeability (FITC-dextran) ↑ Occludin expression ↓ Claudin-5 expression ↓ ROS ↑ NADPH oxydase ↑ ZO-1 expression ↓ IL-6 expression ↑	Rochfort et al., 2014 Rochfort and Cummins, 2015 Rochfort et al., 2016
	Human	Expression of secreted protein acidic and rich in cysteine (SPARC) ↑ Permeability (FITC-dextran) ↑ TEER ↓ ZO-1 expression ↓ Occludin expression ↓	Alkabie et al., 2016
	Human	Expression of cellular prion protein (PrPc) ↓ Permeability (FITC-dextran) ↑	Megra et al., 2018
	Bovine	Permeability (sucrose) ↑ Permeability (inulin) ↑	Deli et al., 1995b
	Bovine	Transcytosis (FITC-holotransferrin) ↑ ERK1/2 signaling ↑	Miller et al., 2005
	Rat	TEER ↓	de Vries et al., 1996
	Rat	Permeability (sodium fluorescein) ↑ TEER ↓ NF-κB signaling ↑	Coelho-Santos et al., 2015
	Rat	Endocytosis (horseradish peroxidase) ↑ PKC signaling ↑	Defazio et al., 2000
	Mouse	Claudin-5 expression ↓ NF-κB signaling ↑	Aslam et al., 2012
	Mouse	Claudin-5 expression ↓ PI3K signaling ↑	Camire et al., 2015
	Mouse	TEER ↓ F-actin rearrangement PKCα/RhoA signaling pathway↑	Peng et al., 2011
Combination of TNF-α and INF-γ	Human	Permeability (FITC-dextran) ↑ Activation of caspase-3 and caspase-9 ↑	Lopez-Ramirez et al., 2012
Combination of TNF-α, IL-6 and IL17	Mouse	Permeability (sodium fluorescein) ↑ TEER ↓ NF-κB transcription factor DNA binding activity ↑	Voirin et al., 2020
IL-6	Human	Permeability (FITC-dextran) ↑ Occludin expression ↓ Claudin-5 expression ↓ ROS ↑	Rochfort et al., 2014
	Mouse	Permeability (sodium fluorescein) ↑ TEER ↓ ZO-1 expression ↓ Claudin-5 expression ↓	Voirin et al., 2020
OSM	Human	Expression of intercellular adhesion molecule-1 ↑ Expression of vascular cell adhesion molecule-1 →	Ruprecht et al., 2001
	Rat	Permeability (sodium fluorescein) ↑ TEER ↓ Claudin-5 expression ↓ JAK/STAT3 signaling pathway ↑	Takata et al., 2008, 2018
**Lipid mediators**			
PGE2	Human	Permeability (FITC-Dextran) ↑ through activation of EP3 and EP4	Dalvi et al., 2015
	Bovine	Permeability (FITC-Dextran) ↑	Mark et al., 2001
TXA2	Human	Occludin expression ↓ Claudin-5 expression ↓ Activate the ROCK-PTEN-Akt-eNOS pathway	Zhao et al., 2017
LPA	Human	Permeability (Texas red-Dextran) ↑ Occludin expression ↓ Claudin-5 expression ↑	Kim et al., 2018
	Rat	TEER ↓ Activate the LPA–LPA6–G12/13–Rho pathway	Masago et al., 2018
	Mouse	TEER ↓ Phosphorylation of occludin and claudin-5 through activation of Rho Kinase ↑	Yamamoto et al., 2008
S1P	Human	TEER ↑ through activation of S1PR5 receptor	van Doorn et al., 2012
	Human	TEER ↑	Alves et al., 2019
	Human	TEER ↓	Wiltshire et al., 2016
	Rat	TEER ↓ Permeability (Sodium fluorescein) ↑	Nakagawa and Aruga, 2020
Serum amyliod A (SAA)	Rat	Permeability (Sodium fluorescein) ↑ TEER ↓ Claudin-5 expression ↓	Matsumoto et al., 2020
**Autoantibody**			
Antibody from patients of NMO (target antigen: glucose-regulated protein 78)	Human	Permeability (FITC-Dextran) ↑ Permeability (IgG) ↑ NF-κB nuclear translocation ↑ Claudin-5 expression ↓	Shimizu et al., 2017
Antibody from patients of PCD with LEMS (target antigen: glucose-regulated protein 78)	Human	Permeability (FITC-Dextran) ↑ TEER ↓ Claudin-5 expression ↓ NF-κB nuclear translocation ↑	Shimizu et al., 2019
Antibody from patients of SPMS (target antigen: galectin-3)	Human	TEER ↓ Claudin-5 expression ↓ ICAM-1 expression ↑	Shimizu et al., 2014; Nishihara et al., 2017

### IL-6

IL-6 induced an increase in BMEC permeability and a decrease in expression of the tight junction proteins claudin-5, occludin, and ZO-1 (Voirin et al., 2020). The decreased expression of tight junction proteins induced by IL-6 was mediated by ROS generation in BMECs (Rochfort et al., 2014). In addition, endogenous IL-6 secreted by BMECs contributed to the dysfunction of barrier integrity in an autocrine manner through its receptor glycoprotein 130 (gp130) (Dohgu et al., 2011; Rochfort et al., 2016). However, Takata et al. (2018) reported that BMECs were less sensitive to IL-6 at the same concentration as OSM, another member of the IL-6 family. This lower sensitivity of BMECs to IL-6 could be explained by the lower activation level of STAT3 induced by IL-6. Although IL-6 itself possesses the ability to induce BBB dysfunction, IL-6 may act as a potentiator of other inflammatory mediators such as TNF-α and IL-17 and/or an inducer of microglial activation (Matsumoto et al., 2014, 2018) (see Table 1).

### Oncostatin M

Oncostatin M is a member of the IL-6 cytokine family. OSM receptors are expressed on BMECs and form heterodimers with gp130 to transduce OSM signaling (Bauer et al., 2007; Takata et al., 2018). When BMECs were treated with OSM, increased BBB permeability, reduced TEER, and decreased expression of claudin-5, a tight junction-associated protein, were observed (Takata et al., 2008, 2018). In MS and TBI associated with neuroinflammation, OSM was elevated in the brain and BBB functions were impaired (Ruprecht et al., 2001; Minagar and Alexander, 2003; Oliva et al., 2012; Logsdon et al., 2018). Taken together, these findings implicate elevated OSM in the brain in the impairment of BBB functions under pathological conditions with neuroinflammation. Our previous study demonstrated that OSM was an intensive molecule for induction of BBB dysfunction *via* prolonged activation of STAT3 following JAK activation (Takata et al., 2018). Although other cytokines belonging to the IL-6 family, IL-6 and LIF, also induced transient activation of STAT3 in BMECs, their activity for downregulation of BBB functions was markedly lower than that of OSM. These findings suggest that prolonged OSM-induced STAT3 activation is largely conducive to impairment of BBB functions. Thus, under pathological conditions accompanied by increased OSM and activation of STAT3 in the brain, impaired BBB functions could allow harmful molecules to penetrate into the brain.

Besides the downregulation of tight junction-associated proteins, the expression of ICAM-1, which controls immune cell trafficking across the BBB, was increased after exposure of human BMECs to OSM (Ruprecht et al., 2001). In peripheral organs, it was reported that neutrophil-derived OSM augmented P-selectin-dependent neutrophil rolling on postcapillary venules through gp130-dependent signaling in endothelial cells (Setiadi et al., 2019). Thus, OSM-activated adhesion molecules may evoke neutrophil penetration across the BBB, contributing to the neurotoxicity process in neuroinflammatory diseases such as MS. Under pathological conditions, elevated OSM in the brain should be considered as a possible causal factor for the development and progression of neuroinflammation arising through penetration of harmful molecules and immune cells in the circulating blood across the BBB (see Table 1).

### Lipid Mediators

**FIGURE 5:**
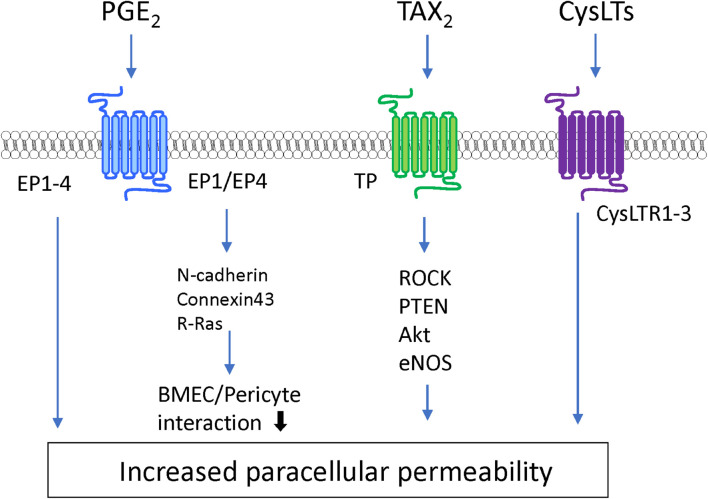
Intracellular signaling pathways induced by eicosanoids in BMECs leading to BBB dysfunction.

**FIGURE 6:**
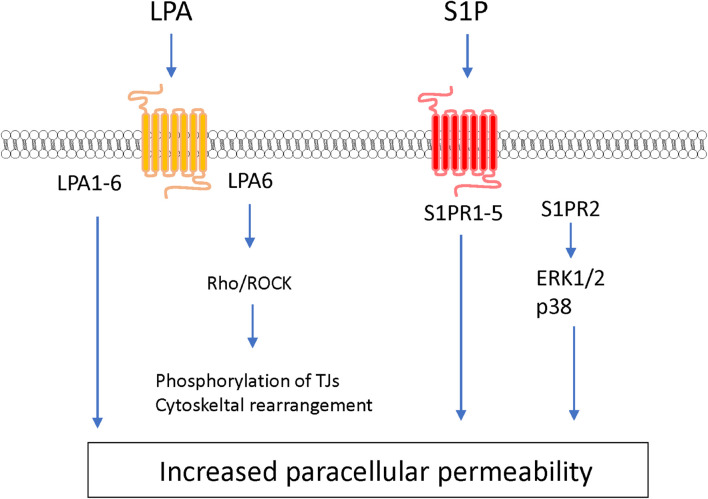
Intracellular signaling pathways induced by lysophosholipids in BMECs leading to BBB dysfunction.

Lipid mediators are bioactive lipids with important roles in many physiological and pathological conditions including inflammation, atherosclerosis, ischemia, and cancer. In response to various stimuli, lipid mediators are rapidly synthesized by specific enzymes. The produced intracellular lipid mediators are secreted into the extracellular space where they act as extracellular signaling molecules like local hormones or autacoids through G protein-coupled receptors (GPCRs). The majority of lipid mediators are produced by multistep enzymatic pathways from lipids that are cellular membrane constituents. These lipid mediators can be classified according to their structures, such as arachidonic acid-derived eicosanoids (e.g., prostaglandins, leukotrienes), lysophospholipids (e.g., lysophosphatidic acid, sphingosine 1-phosphate), and others (Shimizu, 2009; Murakami, 2011). These mediators stimulate the cells that constitute the BBB and modulate BBB functions (see Figures 5, 6 and Table 1).

#### Eicosanoid

Prostanoids and leukotrienes are a subclass of eicosanoids produced from arachidonic acid through continuous enzyme reactions including the cyclooxygenase and lipoxygenase pathways. Among the prostanoids, prostaglandin E2 (PGE_2_), prostaglandin D2 (PGD_2_), prostaglandin F2α (PGF_2α_), prostacyclin (PGI_2_), and thromboxane A2 (TXA_2_) are known to be major bioactive prostanoids. These prostanoids exert their effects by activating their cognate GPCRs (Murakami, 2011). Several studies have indicated that PGE_2_ has the ability to regulate BBB permeability depending on its receptor subtypes (EP1–EP4). PGE_2_ treatment of cultured BMECs increased the permeability to fluorescein-conjugated dextran (Mark et al., 2001). Meanwhile, increased production of PGE_2_ induced by exposure of BMECs to arachidonic acid increased the permeability of BMEC monolayers through activation of EP3 and EP4 (Dalvi et al., 2015). A series of studies involving pharmacological or genetic inhibition of PGE_2_ receptors indicated that EP1 and EP3 contributed to BBB breakdown in ischemic stroke models (Fukumoto et al., 2010; Ikeda-Matsuo et al., 2011; Frankowski et al., 2015). Although EP2 activation in the brain was shown to be a detrimental factor in stroke, neurodegenerative diseases, and status epilepticus (Woodling and Andreasson, 2016; Li et al., 2020; Nagib et al., 2020), the direct effect of EP2 on regulation of BBB functions has not been fully elucidated. Meanwhile, EP4 was reported to play a beneficial role in the brain, including attenuation of the BBB dysfunction induced by stroke (Liang et al., 2011; DeMars et al., 2018). A recent study showed that PGE2-EP1/EP4 interactions induced downregulation of *N*-cadherin, connexin-43, and R-Ras leading to reduced pericyte-BMEC interactions. The impaired interactions between BMECs and pericytes evoked BMEC destabilization and hyperpermeability (Perrot et al., 2020). Prostanoid TXA_2_ also modulated BBB functions. Stimulation of the TXA2 receptor (TP receptor) on BMECs by hyperglycemia or a TP receptor agonist impaired the integrity of the BBB *via* the ROCK-PTEN-Akt-eNOS pathway (Zhao et al., 2017). Meanwhile, a TP receptor antagonist reduced the brain damage induced by ischemic stroke through preserved expression of tight junction proteins and prevention of microglial activation (Yan et al., 2016). Other prostanoids such as PGI_2_, PGD_2_, and PGF_2α_ contributed to modifications of brain function including blood-flow regulation and neuroprotection (Ahmad, 2014; Muramatsu et al., 2015; Mohan et al., 2018; Rojas et al., 2019). However, the direct effects of these prostanoids on BBB functions and their putative cell signaling pathways remain unclear. Therefore, systematic examinations of the effects of prostanoids on the regulation of BBB functions are warranted.

In addition to prostanoids, cysteinyl leukotrienes (CysLTs) act as potent proinflammatory mediators and are considered to modulate BBB functions. CysLTs including LTC_4_, LTD_4_, and LTE_4_ are metabolites of arachidonic acid *via* the lipoxygenase pathway that stimulate cognate receptors named cysteinyl leukotriene receptors (CysLTR1–3) (Shimizu, 2009; Murakami, 2011). Injection of LTC_4_, LTE_4_, or LTB_4_ into the brain parenchyma induced BBB hyperpermeability (Black and Hoff, 1985). Furthermore, studies on various animal models including ischemic stroke, AD, MS, and epilepsy indicated that pharmacological inhibition of CysLTRs had beneficial effects such as improvement of BBB dysfunction, inhibition of microglia and/or astrocyte activation, and inhibition of neuroinflammation (Gelosa et al., 2017; Chen et al., 2020) (see Table 1).

#### Lysophospholipids

As another type of bioactive signaling lipids, lysophospholipids are synthesized from phospholipids and sphingolipids. Lysophospholipids including lysophosphatidic acid (LPA) and sphingosine 1-phosphate (S1P) induce a variety of cellular physiological responses *via* cognate GPCR-mediated signaling pathways.

Lysophosphatidic acid is a bioactive lysophospholipid that induces cellular responses *via* specific GPCRs (LPA1–6). LPA is produced from lysophosphatidylcholine by the action of autotaxin (Hao et al., 2020). Several studies have indicated that LPA treatment of BMECs induces BBB impairment. Masago et al. (2018) reported that LPA stimulation of BMECs induced decreases in TEER in an LPA dose-dependent manner and disrupted the structural integrity of tight junction proteins. The same research group further investigated the putative cell signaling pathway involved in the LPA-induced BBB impairment. The barrier reduction was improved by silencing of LPA6 or treatment with a Rho-associated protein kinase (ROCK) inhibitor (Masago et al., 2018). Other research groups reported that LPA induced phosphorylation of tight junction proteins (claudin-5 and occludin) and cytoskeletal rearrangements *via* the Rho-ROCK pathway (Yamamoto et al., 2008; Kim et al., 2018). Taken together, these findings indicate that LPA stimulates LPA receptors coupled with G_12/13_ to activate the Rho-ROCK pathway, leading to an increase in BBB permeability.

S1P is synthesized by phosphorylation of sphingosine derived from sphingolipids by sphingosine kinases 1 and 2 (Sphk1 and Sphk2). Intracellular S1P is secreted into the extracellular space *via* S1P transporters (e.g., Abca1 and Spns2) where it acts as an extracellular signaling molecule through GPCRs (S1PR1–5) coupled with G_q_, G_i_, G_12/13_, and Rho proteins (Hao et al., 2020; Pluimer et al., 2020). S1P regulates a number of biological processes including vascular development and function. The direct effect of S1P on cultured BMECs remains controversial. Some research groups have reported barrier enhancement following treatment with S1P (van Doorn et al., 2012; Alves et al., 2019), while other research groups found that S1P decreased barrier properties in BMECs (Wiltshire et al., 2016; Nakagawa and Aruga, 2020). Given that S1P has different effects on endothelial barrier function depending on the S1PRs involved (Camm et al., 2014), the differences between these conflicting findings may be caused by altered expression of S1PRs in different cell types and culture conditions. *In vitro* studies using endothelial cells derived from non-brain tissues indicated that S1PR1 strengthens the barrier function, while S1PR2 reduces the barrier function (Camm et al., 2014; Kerage et al., 2014). *In vivo* studies demonstrated that endothelial-specific S1PR1 knockout mice had facilitated small-molecule-selective BBB opening (Yanagida et al., 2017). Conversely, S1PR2 contributed to induction of BBB impairment. Several research groups reported that S1PR2 played a critical role in induction of BBB dysfunction in ischemic stroke and experimental autoimmune encephalomyelitis models (Cruz-Orengo et al., 2014; Kim et al., 2015). Cao et al. (2019) revealed that activation of p38 MAPK and ERK1/2 signaling stimulated by S1P-S1PR2 interactions was involved in oxidative stress-induced BMEC barrier impairment. Another research group indicated that S1PR2 expressed in pericytes contributed to increased pericyte migration and reduced *N*-cadherin expression *via* the NF-κB p65 signaling pathway (Wan et al., 2018). Meanwhile, S1P/S1PR3 signaling mediated proliferation of pericytes *via* the Ras/pERK pathway, which may be involved in scar formation after spinal cord injury (Tang et al., 2018). Finally, stimulation of S1PR3 on astrocytes enhanced expression of inflammatory genes through RhoA signaling (Dusaban et al., 2017).

Much of the evidence regarding the roles of lipid mediators in regulating endothelial barrier functions has been accumulated using non-brain-derived endothelial cells. It is widely accepted that brain capillary endothelial cells have several characteristic differences compared with peripheral endothelial cells. Furthermore, Daneman et al. (2010) indicated that numerous signaling cascades and metabolic pathways in BMECs differed from those in peripheral endothelial cells. Thus, further studies using brain-derived endothelial cells are needed to elucidate the actual roles of lipid mediators in BBB functions (see Table 1).

### Serum Amyloid A

Serum amyloid A (SAA) is a major acute-phase protein and its serum level increases by up to 1,000-fold during acute inflammation (Ye and Sun, 2015). A pivotal role for SAA in mediating the pathological processes of brain disorders has also been identified. SAA was elevated in brain disorders such as cognitive impairment, depression, and ischemic stroke (Shang et al., 2018). SAA was also upregulated in subjects with impaired BBB relative to subjects with intact BBB (Bowman et al., 2018). These findings suggest that SAA may alter BBB integrity and impair neurological function in pathological states. Matsumoto et al. (2020) recently reported that recombinant Apo-SAA impaired brain endothelial integrity associated with decreased claudin-5 protein expression in rat brain endothelial cells (RBECs). The pleiotropic functions of SAA are mediated by several receptors including formyl peptide receptor 2 (FPR2), Toll-like receptor 4 (TLR4), TLR2, CD36, P2X receptor 7, and RAGE (Ye and Sun, 2015). Binding of SAA to multiple SAA receptors simultaneously activates several intracellular signaling pathways including the PI3K/Akt, MAPK, and NF-κB signaling pathways (Ye and Sun, 2015; Yu et al., 2017; Sack, 2018). Activation of these signaling pathways was involved in the loss of claudin-5 in endothelial cells (András et al., 2005; Schreibelt et al., 2007; Huang et al., 2016). Apo-SAA induced phosphorylation of NF-κB and p38 MAPK in RBECs (Matsumoto et al., 2020). Furthermore, co-treatment with SAA and high-density lipoprotein (HDL), an apolipoprotein of SAA in circulating blood, recovered the brain endothelial barrier function and decreased claudin-5 expression in Apo-SAA-treated RBECs. In addition, HDL also reduced the phosphorylation of NF-κB and p38 MAPK in the treated cells (Matsumoto et al., 2020). These findings suggest that SAA may downregulate claudin-5 expression *via* multiple signaling pathways, leading to brain endothelial barrier dysfunction (see Table 1).

### Autoantibody

Blood-brain barrier dysfunction has been reported in several immune-mediated neuroinflammatory diseases, including neuromyelitis optica (NMO) and multiple sclerosis (MS). Accumulating evidence has indicated that autoantibodies in patients with these diseases directly mediate BBB dysfunction (Shimizu et al., 2018; Table 1). IgG from secondary progressive MS (SPMS-IgG) sera induced decreased TEER and claudin-5 expression, as well as increased VCAM-1 expression in human BMECs (Shimizu et al., 2014). One previous study reported that the target molecule of SPMS-IgG for inducing BBB dysfunction was galectin-3, and galectin-3 antibodies in the SPMS sera influenced NF-κB p65 signaling (Nishihara et al., 2017). These findings suggested that galectin-3 autoantibodies were the factors responsible for inducing BBB dysfunction in progressive MS. NMO patient-derived IgG (NMO-IgG) caused the hyperpermeability and decreased levels of claudin-5 expressions through NF-κB nuclear translocation in human BMECs (Shimizu et al., 2017). In addition, the absorption of GRP78-specific antibodies from NMO-IgG resulted in decreased nuclear translocation of NF-κB in human BMECs, suggesting that GRP78 autoantibodies directly induce BBB dysfunction in BMECs. Besides NMO-IgG, IgG derived from patients of paraneoplastic cerebellar degeneration (PCD) with the autoimmune disease Lambert-Eaton myasthenic syndrome (LEMS) was found to impaired the barrier integrity in human BMECs (Shimizu et al., 2019). NF-κB nuclear translocation resulting in BBB dysfunction was inhibited when GRP78-specific IgG was removed from PCD-LEMS IgG. Based on these data, identifying autoantibodies mediating BBB dysfunction might lead to the discovery of target molecules in BMEC to regulate BBB function under immune-mediated neurological diseases (see Table 1).

### MicroRNAs

**FIGURE 7:**
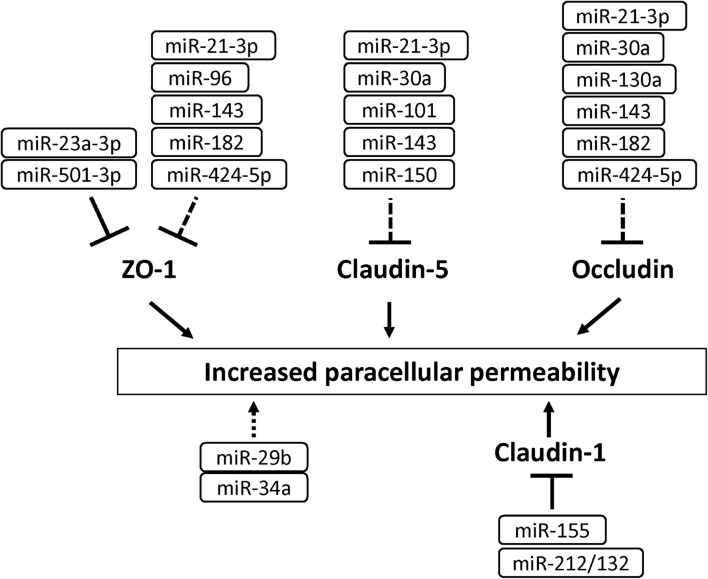
MicroRNAs involved in the down-regulation of tight junction protein levels and BBB function in BMECs. Solid and dashed lines indicate that miRNAs directly and indirectly down-regulate tight junction-associated proteins, respectively.

MicroRNAs (miRNAs) are small non-coding RNA (21–25 nucleotides) and inhibit translation of target mRNA by binding to 3′ untranslated regions. Several miRNAs have been shown to play a critical role in the development of BBB dysfunction in CNS diseases involving neuroinflammation, such as stroke, TBI, dementia and cerebral infections (Ma et al., 2020). Supplementary Table 1 shows miRNAs associated with the downregulation of BBB integrity in BMECs (Bukeirat et al., 2016; Fang et al., 2016; Hu et al., 2018; Toyama et al., 2018). Under ischemic conditions, the expression levels of miR-212/132, -21-3p, -30a, -130a, and -182 were increased in BMECs, and these miRNAs induced hyperpermeability, decreased TEER and downregulated levels of tight junction-associated proteins in BMECs (Wang et al., 2018, 2021; Burek et al., 2019; Ge et al., 2019; Zhang et al., 2020). The increased miR-424-5p in BMECs treated with Aβ induced decreased TEER and downregulated levels of ZO-1 and occludin in BMECs (Lin et al., 2019). Stimulation of BMECs with homocysteine, HIV-1 Tat C, cytokine, or methamphetamine elicited the downregulation of BBB integrity through the modulation of miR-29b, -96, -101 or -143 levels in BMECs, respectively (Mishra and Singh, 2013; Kalani et al., 2014; Bai et al., 2016; Zhang et al., 2018). Based on these data, the regulation of miRNA as critical therapeutic targets should be considered to maintain BBB function under pathological conditions (see Figure 7 and Supplementary Table 1).

### Long Non-coding RNAs

**FIGURE 8:**
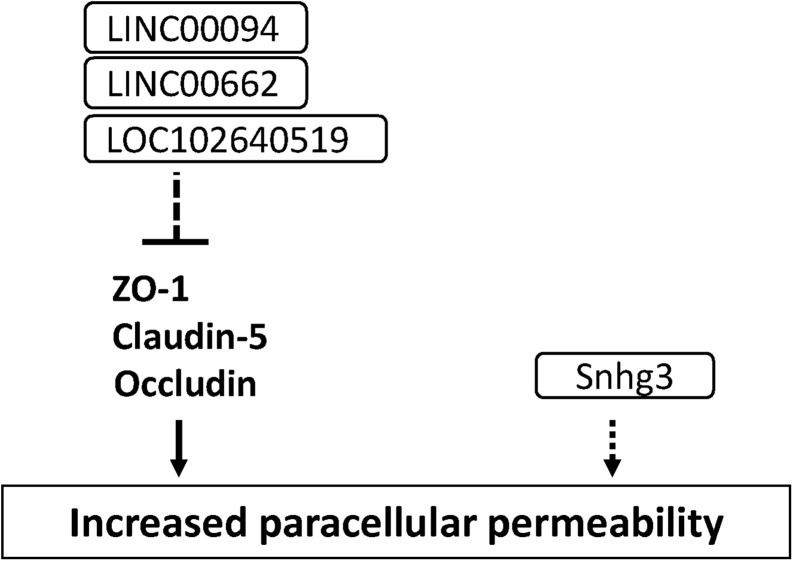
Long non-coding RNAs involved in the down-regulation of tight junction protein levels and BBB function in BMECs. Dashed line indicates indirect down-regulation by lncRNAs. Dashed arrow indicates unknown mechanisms.

Long non-coding RNAs (lncRNAs) are defined as RNA transcripts with lengths exceeding 200 nucleotides that do not encode proteins (Quinn and Chang, 2016). Lnc RNAs affect various molecular events including gene transcription, translation, splicing and protein interaction. Accumulating studies have demonstrated that lncRNAs induced cerebral endothelial pathology which is associated with stroke and Alzheimer’s disease (Yin et al., 2014; Zhang et al., 2016). Increased LINC00094 and LINC00662 in amyloid-β-treated BMECs mediated BBB dysfunction through the down-regulation of tight junction-associated proteins (Zhu et al., 2019; Liu Q. et al., 2020). The increased lncRNA small nucleolar RNA host gene 3 (Snhg3) levels in BMECs led to the BBB hyperpermeability under the condition of intracerebral hemorrhage (Zhang et al., 2019). The increased LOC102640519 expression in BMECs negatively regulated the expressions of ZO-1, claudin-5 and occludin in ischemic conditions (Wu et al., 2018). These findings suggest that lncRNA is a possible therapeutic target for treating the impaired BBB under the pathological conditions (see Figure 8 and Supplementary Table 1).

## Signaling Pathways in BMECs That Strengthen BBB Integrity

### AMPK

5′-Adenosine monophosphate-activated protein kinase (AMPK) is a signaling molecule that regulates BBB functions. Activation of AMPK in BMECs induced upregulation of BBB integrity, featuring increased TEER and decreased BBB permeability to tracers such as sodium fluorescein and Evans blue (Takata et al., 2013). Exposure of mice to LPS, a mediator of neuroinflammation, evoked BBB extravasation of IgG and Evans blue and decreased expression of tight junction proteins including claudin-5 through decreased AMPK activation (Wang et al., 2017). Reduced AMPK signaling in BMECs is likely to be involved in BBB impairment under neuroinflammatory conditions. Indeed, activation of AMPK by metformin, AICAR, and melatonin alleviated the impaired BBB functions under pathological conditions in mouse models of ischemic stroke and sepsis (Wang et al., 2017). Considering these findings, regulation of AMPK activity in BMECs is a therapeutic target for restoring impaired BBB functions in neuroinflammatory diseases (see Table 2).

**TABLE 2:** Signaling pathways in BMECs that strengthen BBB integrity.

Signaling molecules	Species	Cellular events in BMECs	References
AMPK	Rat	AMPK activation ↑ Permeability (sodium fluorescein) ↓ Permeability (Evans blue albumin) ↓ TEER ↑	Takata et al., 2013
cAMP	Bovine	cAMP ↑ TEER ↑	Rubin et al., 1991
	Bovine	cAMP ↑ Permeability (sucrose) ↓ Permeability (inulin) ↓	Deli et al., 1995a
	Porcine	cAMP ↑ TEER ↑ Claudin-5 expression ↑ cAMP/PKA signaling pathway ↑ Phosphorylation of claudin-5 ↑	Ishizaki et al., 2003
Wnt/β-catenin	Human	Wnt/β-catenin signaling pathway ↑ Permeability (Lucifer Yellow salt) ↓ Claudin-5 expression ↑ Claudin-3 expression ↑	Paolinelli et al., 2013
	Human	Wnt signaling ↑ β-catenin expression ↑ Permeability (sodium fluorescein) ↓ Electrical impedance ↑ Claudin-1 expression ↑	Laksitorini et al., 2019

### cAMP

Intracellular cyclic adenosine monophosphate (cAMP) elevation is a well-known factor that induces strong barrier properties in endothelial cells. Treatment of cultured BMECs with a cell-permeable cAMP analog, adenylate cyclase activator, and a phosphodiesterase inhibitor increased the barrier function, by increasing TEER and decreasing paracellular permeability (Rubin et al., 1991; Deli et al., 1995a, 2005). Ishizaki et al. (2003) found that cAMP acted on tight junctions in the BBB to increase expression of claudin-5 in a protein kinase A (PKA)-independent manner and phosphorylated claudin-5 *via* the cAMP/PKA pathway to strengthen barrier tightness (Ishizaki et al., 2003). Other research groups found that activation of an exchange protein directly activated by cAMP1 (Epac1), a downstream effector of cAMP, protected barrier integrity in peripheral vascular endothelial cells (Fukuhara et al., 2005; Lorenowicz et al., 2008). Therefore, the cAMP/PKA and cAMP/Epac1 signaling pathways are considered to independently impact on barrier integrity in endothelial cells. Importantly, a deterioration effect of cAMP on barrier function was reported by several research groups, indicating that cAMP produced in the cytosol by activation of soluble adenylyl cyclases plays a role in the disruption of barrier function (Sayner et al., 2004; Prasain et al., 2009; Sayner, 2011). Therefore, cAMP produced in the cytosol and cAMP produced by membrane-associated enzymes seem to have opposite effects on endothelial barrier stabilization. Although the roles of cAMP in BMECs, such as reduction of intracellular cAMP and site-specific production of cAMP, have been not fully elucidated, cAMP signaling is one of the important pathways for modulation of BBB functions (see Table 2).

### Wnt/β-Catenin

Wnt/β-catenin signaling is important for brain vascularization and BBB differentiation. Dysregulation of the Wnt/β-catenin pathway is observed in various CNS diseases associated with BBB dysfunction. Wnt can activate both the canonical Wnt/β-catenin signaling pathway and the non-canonical Wnt pathway, which is divided into Wnt/planar cell polarity and Wnt/Ca^2+^ utilization pathways. BMECs expressed Wnt receptor (Frizzled), co-receptors (LRP5, LRP6, ROR2, and RYK), Wnt ligands, and Wnt modulator peptides (DKK, sFRP, and WIF) (Laksitorini et al., 2019). Activation of Wnt/β-catenin signaling in BMECs following treatment with Wnt ligand Wnt3a, which is not expressed in BMECs, or LiCl, increased β-catenin and mRNA expression of claudin-5 and occludin to tighten the paracellular barrier. LiCl is a GSK3β inhibitor that inhibits β-catenin phosphorylation and degradation (Paolinelli et al., 2013). Although Wnt ligands are also expressed in perivascular cells, pericytes, and astrocytes to regulate and maintain the BBB, modulation of brain endothelial Wnt activation is a possible pathway for restoration of BBB functions (see Table 2).

## Conclusion and Perspectives

In this review, we focused on the understanding of cellular events in BMECs that lead to BBB dysfunction because protection of the BBB would be a therapeutic target for the development of neurodegenerative diseases involving neuroinflammation. Although a large body of evidence from *in vivo* studies suggests that various inflammatory mediators released by neurovascular unit cells surrounding BMECs induce BBB dysfunction, only a limited number of *in vitro* studies have addressed the direct BBB-impairing effects of inflammatory mediators derived from neurovascular unit cells using co-culture systems with BMECs. More work is needed to identify secreted inflammatory mediators including non-coding RNAs and their related intracellular signaling pathways involved in BBB dysfunction.

In this context, therapeutic drugs for BBB protection need to possess the following abilities: (1) strengthening of brain endothelial barrier properties directly, (2) blockage of intracellular signaling pathways in BMECs leading to BBB dysfunction, and (3) inhibition of release of inflammatory mediators from neurovascular unit cells, particularly pericytes, astrocytes and microglia. Many efforts have been made to discover BBB protective compounds including pharmacological inhibitors of signaling pathways and natural compounds. However, none of these are currently approved therapeutic drugs. Therefore, drug repositioning is an effective approach to discover BBB protective drugs. Table 3 shows a list of possible candidates from *in vitro* studies demonstrating an improved and/or strengthening effect on barrier properties of BMECs (Table 3). *In vivo* studies were excluded because it is unclear whether administrated drugs directly affect neurovascular units. Metformin (Takata et al., 2013), pitavastatin (Morofuji et al., 2010), siponimod (Spampinato et al., 2021), and cilostazol (Horai et al., 2013) possess the ability to strengthen brain endothelial barrier properties through modulating intracellular signaling in physiological conditions. Minocycline (Yang et al., 2015), alogliptin (Hao et al., 2019), zafirlukast (Zeng et al., 2020), siponimod (Spampinato et al., 2021), memantine (Zhu et al., 2019), cilostazol (Horai et al., 2013; Takeshita et al., 2014), candesartan (So et al., 2015), perampanel (Chen et al., 2021), lithium (Ji et al., 2021), omarigliptin (Du and Wang, 2020) and 5′-azacytidine (Kalani et al., 2014) restore the barrier function dependently of specific pathological conditions (e.g., hypoxia, etc.). Ruxolitinib (Takata et al., 2019) inhibits activation of pericytes to release inflammatory mediators, resulting in restoration of the BBB. Propofol (Sun et al., 2019) protects hypoxia-mediated BBB impairment through regulating microglia and astrocytes. BMECs are the interface between the circulating blood and the brain parenchyma. This location is advantageous for pharmacological interventions targeting BMECs because it is not necessary to consider the ability of therapeutic compounds to cross the BBB. Therefore, to navigate and accelerate drug repositioning by high-throughput screening, further studies are needed to determine the intracellular signaling pathways in BMECs that lead to BBB dysfunction and/or strengthening of BBB integrity. New supportive approaches to treat neurodegenerative diseases with neuroinflammation could potentially be developed by targeting (1) maintenance of BBB integrity by blockade of intracellular signaling pathways in BMECs that lead to BBB dysfunction and (2) repair of a leaky BBB by stimulating BMECs to activate signaling pathways that strengthen the barrier integrity.

**TABLE 3:** Therapeutic drug candidates for modulating BBB integrity.

Therapeutic drug candidates	Species	Cocultured cells with BMECs	Experimental conditions	Cellular events in BMECs	References
Minocycline	Human	-	Oxygen deprivation	TEER ↑ Claudin-5 expression ↑ Occludin expression ↑ ZO-1 expression ↑	Yang et al., 2015
Alogliptin	Human	-	OGD/reoxygenation	Permeability (FITC-Dextran) ↓ Occludin expression ↑ ZO-1 expression ↑	Hao et al., 2019
Zafirlukast	Human	-	OGD/reoxygenation	Permeability (FITC-Dextran) ↓ Occludin expression ↑ ZO-1 expression ↑	Zeng et al., 2020
Siponimod	Human	Astrocytes	Physiological conditions	Permeability (FITC-Dextran) ↓ Claudin-5 expression ↑ ZO-1 expression ↑	Spampinato et al., 2021
		Astrocytes	TNF-α and INF-γ treatment	PI3K/Akt signaling pathway↑ Permeability (FITC-Dextran) ↓ TEER ↑ Claudin-5 expression ↑ ZO-1 expression ↑	Spampinato et al., 2021
Memantine	Human	Pericytes and astrocytes	Amyloid-β_1–42_ treatment	LINC00094 expression ↓ Permeability (horseradish peroxidase) ↓ TEER ↑ Claudin-5 expression ↑ Occludin expression ↑ ZO-1 expression ↑	Zhu et al., 2019
Metformin	Rat	-	Physiological conditions	AMPK activation ↑ Permeability (sodium fluorescein) ↓ Permeability (Evans blue albumin) ↓ TEER ↑	Takata et al., 2013
Pitavastatin	Rat	-	Physiological conditions	Mevalonate pathway ↓ Permeability (sodium fluorescein) ↓ Permeability (Evans blue albumin) → TEER ↑ Claudin-5 expression ↑	Morofuji et al., 2010
Cilostazol	Rat	-	Physiological conditions	cAMP↑ Permeability (sodium fluorescein) ↓ TEER ↑	Horai et al., 2013
	Rat	Pericytes and astrocytes	OGD/reoxygenation	TEER ↑	Horai et al., 2013
	Rat	Pericytes and astrocytes	TGF-β1 treatment	TEER ↑	Takeshita et al., 2014
	Rat	Pericytes and astrocytes	AGEs treatment under OGD/reoxygenation	TGF-β1 ↓ TEER ↑ Claudin-5 expression ↑	Takeshita et al., 2014
Candesartan	Rat	Astrocytes	OGD/reoxygenation	Permeability (sodium fluorescein) ↓ TEER ↑ Improved localization of claudin-5 and occludin	So et al., 2015
Perampanel	Rat	Astrocytes and neurons	Glutamate treatment	Permeability (sodium fluorescein) ↓ TEER ↑	Chen et al., 2020
	Rat	Astrocytes and neurons	Traumatic neuronal injury model	Permeability (sodium fluorescein) ↓ TEER ↑	Chen et al., 2020
Ruxolitinib	Rat	Pericytes	OSM treatment	Permeability (sodium fluorescein) ↓ TEER ↑ (JAK/STAT3 signaling pathway in pericytes ↓)	Takata et al., 2019
Lithium	Mouse	-	OGD/reoxygenation	Wnt/β-catenin signaling pathway ↑ TEER ↑	Ji et al., 2021
Omarigliptin	Mouse	-	LPS treatment	Toll-like receptor 4/myeloid differentiation factor 88/NF-κB signaling pathway ↓ Permeability (FITC-Dextran) ↓ Claudin-1 expression ↑ Claudin-5 expression ↑	Du and Wang, 2020
Propofol	Mouse	Astrocytes and microglia	Oxygen deprivation	TEER ↑ (Heat shock protein (HSP) 32 expressions and nuclear translocation of nuclear factor-E2-related factor 2 in microglia ↑) (HSP27 expressions and nuclear translocation of heat shock factor 1 in astrocytes ↑)	Sun et al., 2019
5′-azacytidine	Mouse	-	Homocysteine treatment	miR-29b expression ↓ Permeability (FITC-albumin) ↓	Kalani et al., 2014

## Author Contributions

All authors listed have made a substantial, direct and intellectual contribution to the work, and approved it for publication.

## Conflict of Interest

The authors declare that the research was conducted in the absence of any commercial or financial relationships that could be construed as a potential conflict of interest.

## Publisher’s Note

All claims expressed in this article are solely those of the authors and do not necessarily represent those of their affiliated organizations, or those of the publisher, the editors and the reviewers. Any product that may be evaluated in this article, or claim that may be made by its manufacturer, is not guaranteed or endorsed by the publisher.

## References

[B1] Ahmad A. S. (2014). PGD2 DP1 receptor stimulation following stroke ameliorates cerebral blood flow and outcomes. *Neuroscience* 279 260–268. 10.1016/j.neuroscience.2014.08.050 25218962

[B2] Al-Bachari S. Naish J. H. Parker G. J. M. Emsley H. C. A. Parkes L. M. (2020). Blood-brain barrier leakage is increased in Parkinson’s disease. *Front. Physiol.* 11:593026. 10.3389/fphys.2020.593026 33414722 PMC7784911

[B3] Alkabie S. Basivireddy J. Zhou L. Roskams J. Rieckmann P. Quandt J. A. (2016). SPARC expression by cerebral microvascular endothelial cells in vitro and its influence on blood-brain barrier properties. *J. Neuroinflammation* 13:225. 10.1186/s12974-016-0657-9 PMC5007716 27581191

[B4] Alvarez J. I. Cayrol R. Prat A. (2011). Disruption of central nervous system barriers in multiple sclerosis. *Biochim. Biophys. Acta* 1812 252–264. 20619340 10.1016/j.bbadis.2010.06.017

[B5] Alves N. G. Yuan S. Y. Breslin J. W. (2019). Sphingosine-1-phosphate protects against brain microvascular endothelial junctional protein disorganization and barrier dysfunction caused by alcohol. *Microcirculation* 26:e12506. 10.1111/micc.12506 30281888 PMC6335152

[B6] András I. E. Deli M. A. Veszelka S. Hayashi K. Hennig B. Toborek M. (2007). The NMDA and AMPA/KA receptors are involved in glutamate-induced alterations of occludin expression and phosphorylation in brain endothelial cells. *J. Cereb. Blood Flow Metab.* 27 1431–1443. 10.1038/sj.jcbfm.9600445 17245419

[B7] András I. E. Pu H. Tian J. Deli M. A. Nath A. Hennig B. (2005). Signaling mechanisms of HIV-1 Tat-induced alterations of claudin-5 expression in brain endothelial cells. *J. Cereb. Blood Flow Metab.* 25 1159–1170. 10.1038/sj.jcbfm.9600115 15815581

[B8] Aslam M. Ahmad N. Srivastava R. Hemmer B. (2012). TNF-alpha induced NFkappaB signaling and p65 (RelA) overexpression repress Cldn5 promoter in mouse brain endothelial cells. *Cytokine* 57 269–275. 10.1016/j.cyto.2011.10.016 22138107

[B9] Bai Y. Zhang Y. Hua J. Yang X. Zhang X. Duan M. (2016). Silencing microRNA-143 protects the integrity of the blood-brain barrier: implications for methamphetamine abuse. *Sci. Rep.* 6:35642. 10.1038/srep35642 PMC5073292 27767041

[B10] Bauer A. T. Burgers H. F. Rabie T. Marti H. H. (2010). Matrix metalloproteinase-9 mediates hypoxia-induced vascular leakage in the brain via tight junction rearrangement. *J. Cereb. Blood Flow Metab.* 30 837–848. 10.1038/jcbfm.2009.248 19997118 PMC2949161

[B11] Bauer S. Kerr B. J. Patterson P. H. (2007). The neuropoietic cytokine family in development, plasticity, disease and injury. *Nat. Rev. Neurosci.* 8 221–232. 10.1038/nrn2054 17311007

[B12] Beard R. S. Jr. Haines R. J. Wu K. Y. Reynolds J. J. Davis S. M. Elliott J. E. (2014). Non-muscle Mlck is required for beta-catenin- and FoxO1-dependent downregulation of Cldn5 in IL-1beta-mediated barrier dysfunction in brain endothelial cells. *J. Cell Sci.* 127 1840–1853. 10.1242/jcs.144550 24522189 PMC4074294

[B13] Beers D. R. Appel S. H. (2019). Immune dysregulation in amyotrophic lateral sclerosis: mechanisms and emerging therapies. *Lancet Neurol.* 18 211–220. 10.1016/s1474-4422(18)30394-6 30663610

[B14] Bell R. D. Winkler E. A. Sagare A. P. Singh I. LaRue B. Deane R. (2010). Pericytes control key neurovascular functions and neuronal phenotype in the adult brain and during brain aging. *Neuron* 68 409–427. 10.1016/j.neuron.2010.09.043 21040844 PMC3056408

[B15] Black K. L. Hoff J. T. (1985). Leukotrienes increase blood-brain barrier permeability following intraparenchymal injections in rats. *Ann. Neurol.* 18 349–351. 10.1002/ana.410180313 2996417

[B16] Blamire A. M. Anthony D. C. Rajagopalan B. Sibson N. R. Perry V. H. Styles P. (2000). Interleukin-1beta -induced changes in blood-brain barrier permeability, apparent diffusion coefficient, and cerebral blood volume in the rat brain: a magnetic resonance study. *J. Neurosci.* 20 8153–8159. 10.1523/jneurosci.20-21-08153.2000 11050138 PMC6772751

[B17] Bowman G. L. Dayon L. Kirkland R. Wojcik J. Peyratout G. Severin I. C. (2018). Blood-brain barrier breakdown, neuroinflammation, and cognitive decline in older adults. *Alzheimers Dement* 14 1640–1650. 10.1016/j.jalz.2018.06.2857 30120040

[B18] Bowman G. L. Kaye J. A. Moore M. Waichunas D. Carlson N. E. Quinn J. F. (2007). Blood-brain barrier impairment in Alzheimer disease: stability and functional significance. *Neurology* 68 1809–1814. 10.1212/01.wnl.0000262031.18018.1a 17515542 PMC2668699

[B19] Bukeirat M. Sarkar S. N. Hu H. Quintana D. D. Simpkins J. W. Ren X. (2016). MiR-34a regulates blood-brain barrier permeability and mitochondrial function by targeting cytochrome c. *J. Cereb. Blood Flow Metab.* 36 387–392. 10.1177/0271678x15606147 26661155 PMC4759667

[B20] Burek M. König A. Lang M. Fiedler J. Oerter S. Roewer N. (2019). Hypoxia-induced MicroRNA-212/132 alter blood-brain barrier integrity through inhibition of tight junction-associated proteins in human and mouse brain microvascular endothelial cells. *Transl. Stroke Res.* 10 672–683. 10.1007/s12975-018-0683-2 30617994 PMC6842347

[B21] Camire R. B. Beaulac H. J. Willis C. L. (2015). Transitory loss of glia and the subsequent modulation in inflammatory cytokines/chemokines regulate paracellular claudin-5 expression in endothelial cells. *J. Neuroimmunol.* 284 57–66. 10.1016/j.jneuroim.2015.05.008 26025059 PMC4451216

[B22] Camm J. Hla T. Bakshi R. Brinkmann V. (2014). Cardiac and vascular effects of fingolimod: mechanistic basis and clinical implications. *Am. Heart J.* 168 632–644. 10.1016/j.ahj.2014.06.028 25440790

[B23] Cao C. Dai L. Mu J. Wang X. Hong Y. Zhu C. (2019). S1PR2 antagonist alleviates oxidative stress-enhanced brain endothelial permeability by attenuating p38 and Erk1/2-dependent cPLA2 phosphorylation. *Cell Signal* 53 151–161. 10.1016/j.cellsig.2018.09.019 30290210

[B24] Caruso R. A. Fedele F. Finocchiaro G. Pizzi G. Nunnari M. Gitto G. (2009). Ultrastructural descriptions of pericyte/endothelium peg-socket interdigitations in the microvasculature of human gastric carcinomas. *Anticancer Res.* 29 449–453. 19331185

[B25] Chan Y. Chen W. Wan W. Chen Y. Li Y. Zhang C. (2018). Aβ(1-42) oligomer induces alteration of tight junction scaffold proteins via RAGE-mediated autophagy in bEnd.3 cells. *Exp. Cell Res.* 369 266–274. 10.1016/j.yexcr.2018.05.025 29856989

[B26] Chen F. Ghosh A. Lin J. Zhang C. Pan Y. Thakur A. (2020). 5-lipoxygenase pathway and its downstream cysteinyl leukotrienes as potential therapeutic targets for Alzheimer’s disease. *Brain Behav. Immun.* 88 844–855. 10.1016/j.bbi.2020.03.022 32222525

[B27] Chen S. L. Deng Y. Y. Wang Q. S. Han Y. L. Jiang W. Q. Fang M. (2017). Hypertonic saline protects brain endothelial cells against hypoxia correlated to the levels of epidermal growth factor receptor and interleukin-1beta. *Medicine (Baltimore)* 96:e5786. 10.1097/md.0000000000005786 28072729 PMC5228689

[B28] Chen T. Liu W. B. Qian X. Xie K. L. Wang Y. H. (2021). The AMPAR antagonist perampanel protects the neurovascular unit against traumatic injury via regulating Sirt3. *CNS Neurosci. Ther.* 27 134–144. 10.1111/cns.13580 33421349 PMC7804923

[B29] Chitnis T. Weiner H. L. (2017). CNS inflammation and neurodegeneration. *J. Clin. Invest.* 127 3577–3587. 10.1172/jci90609 28872464 PMC5617655

[B30] Coelho-Santos V. Leitao R. A. Cardoso F. L. Palmela I. Rito M. Barbosa M. (2015). The TNF-alpha/NF-kappaB signaling pathway has a key role in methamphetamine-induced blood-brain barrier dysfunction. *J. Cereb. Blood Flow Metab.* 35 1260–1271. 10.1038/jcbfm.2015.59 25899299 PMC4528012

[B31] Correale J. Gaitán M. I. Ysrraelit M. C. Fiol M. P. (2017). Progressive multiple sclerosis: from pathogenic mechanisms to treatment. *Brain* 140 527–546. 27794524 10.1093/brain/aww258

[B32] Corrigan F. Mander K. A. Leonard A. V. Vink R. (2016). Neurogenic inflammation after traumatic brain injury and its potentiation of classical inflammation. *J. Neuroinflammation* 13:264. 10.1186/s12974-016-0738-9 PMC5057243 27724914

[B33] Cruz-Orengo L. Daniels B. P. Dorsey D. Basak S. A. Grajales-Reyes J. G. McCandless E. E. (2014). Enhanced sphingosine-1-phosphate receptor 2 expression underlies female CNS autoimmunity susceptibility. *J. Clin. Invest.* 124 2571–2584. 10.1172/jci73408 24812668 PMC4089451

[B34] Cuevas E. Rosas-Hernandez H. Burks S. M. Ramirez-Lee M. A. Guzman A. Imam S. Z. (2019). Amyloid Beta 25-35 induces blood-brain barrier disruption in vitro. *Metab. Brain Dis.* 34 1365–1374. 10.1007/s11011-019-00447-8 31267346

[B35] da Fonseca A. C. Matias D. Garcia C. Amaral R. Geraldo L. H. Freitas C. (2014). The impact of microglial activation on blood-brain barrier in brain diseases. *Front. Cell Neurosci.* 8:362. 10.3389/fncel.2014.00362 25404894 PMC4217497

[B36] Dalvi S. Nguyen H. H. On N. Mitchell R. W. Aukema H. M. Miller D. W. (2015). Exogenous arachidonic acid mediates permeability of human brain microvessel endothelial cells through prostaglandin E2 activation of EP3 and EP4 receptors. *J. Neurochem.* 135 867–879. 10.1111/jnc.13117 25865705

[B37] Daneman R. Zhou L. Agalliu D. Cahoy J. D. Kaushal A. Barres B. A. (2010). The mouse blood-brain barrier transcriptome: a new resource for understanding the development and function of brain endothelial cells. *PLoS One* 5:e13741. 10.1371/journal.pone.0013741 21060791 PMC2966423

[B38] de Vries H. E. Blom-Roosemalen M. C. van Oosten M. de Boer A. G. van Berkel T. J. Breimer D. D. (1996). The influence of cytokines on the integrity of the blood-brain barrier in vitro. *J. Neuroimmunol.* 64 37–43. 10.1016/0165-5728(95)00148-4 8598388

[B39] Defazio G. Nico B. Trojano M. Ribatti D. Giorelli M. Ricchiuti F. (2000). Inhibition of protein kinase C counteracts TNFalpha-induced intercellular adhesion molecule 1 expression and fluid phase endocytosis on brain microvascular endothelial cells. *Brain Res* 863 245–248. 10.1016/s0006-8993(00)02127-2 10773213

[B40] Deli M. A. Abraham C. S. Kataoka Y. Niwa M. (2005). Permeability studies on in vitro blood-brain barrier models: physiology, pathology, and pharmacology. *Cell. Mol. Neurobiol.* 25 59–127. 10.1007/s10571-004-1377-8 15962509 PMC11529645

[B41] Deli M. A. Descamps L. Dehouck M. P. Cecchelli R. Joó F. Abrahám C. S. (1995b). Exposure of tumor necrosis factor-alpha to luminal membrane of bovine brain capillary endothelial cells cocultured with astrocytes induces a delayed increase of permeability and cytoplasmic stress fiber formation of actin. *J. Neurosci. Res.* 41 717–726. 10.1002/jnr.490410602 7500373

[B42] Deli M. A. Dehouck M. P. Abrahám C. S. Cecchelli R. Joó F. (1995a). Penetration of small molecular weight substances through cultured bovine brain capillary endothelial cell monolayers: the early effects of cyclic adenosine 3′,5′-monophosphate. *Exp. Physiol.* 80 675–678. 10.1113/expphysiol.1995.sp003877 7576606

[B43] DeMars K. M. McCrea A. O. Siwarski D. M. Sanz B. D. Yang C. Candelario-Jalil E. (2018). Protective effects of L-902,688, a prostanoid EP4 receptor agonist, against acute blood-brain barrier damage in experimental ischemic stroke. *Front. Neurosci.* 12:89. 10.3389/fnins.2018.00089 29527151 PMC5829545

[B44] Dohgu S. Fleegal-DeMotta M. A. Banks W. A. (2011). Lipopolysaccharide-enhanced transcellular transport of HIV-1 across the blood-brain barrier is mediated by luminal microvessel IL-6 and GM-CSF. *J. Neuroinflammation* 8:167. 10.1186/1742-2094-8-167 PMC3260201 22129063

[B45] Dohgu S. Takata F. Matsumoto J. Kimura I. Yamauchi A. Kataoka Y. (2019). Monomeric α-synuclein induces blood-brain barrier dysfunction through activated brain pericytes releasing inflammatory mediators in vitro. *Microvasc. Res.* 124 61–66. 30885616 10.1016/j.mvr.2019.03.005

[B46] Du H. Wang S. (2020). Omarigliptin mitigates lipopolysaccharide-induced neuroinflammation and dysfunction of the integrity of the blood-brain barrier. *ACS Chem. Neurosci.* 11 4262–4269. 10.1021/acschemneuro.0c00537 33237730

[B47] Dusaban S. S. Chun J. Rosen H. Purcell N. H. Brown J. H. (2017). Sphingosine 1-phosphate receptor 3 and RhoA signaling mediate inflammatory gene expression in astrocytes. *J. Neuroinflammation* 14:111. 10.1186/s12974-017-0882-x PMC5455202 28577576

[B48] Ensoli F. Fiorelli V. Lugaresi A. Farina D. De Cristofaro M. Collacchi B. (2002). Lymphomononuclear cells from multiple sclerosis patients spontaneously produce high levels of oncostatin M, tumor necrosis factors alpha and beta, and interferon gamma. *Mult. Scler.* 8 284–288. 10.1191/1352458502ms817oa 12166497

[B49] Fang Z. He Q. W. Li Q. Chen X. L. Baral S. Jin H. J. (2016). MicroRNA-150 regulates blood-brain barrier permeability via Tie-2 after permanent middle cerebral artery occlusion in rats. *FASEB J.* 30 2097–2107. 10.1096/fj.201500126 26887441

[B50] Fellner L. Irschick R. Schanda K. Reindl M. Klimaschewski L. Poewe W. (2013). Toll-like receptor 4 is required for α-synuclein dependent activation of microglia and astroglia. *Glia* 61 349–360. 10.1002/glia.22437 23108585 PMC3568908

[B51] Fernández-Klett F. Potas J. R. Hilpert D. Blazej K. Radke J. Huck J. (2013). Early loss of pericytes and perivascular stromal cell-induced scar formation after stroke. *J. Cereb. Blood Flow Metab.* 33 428–439. 10.1038/jcbfm.2012.187 23250106 PMC3587816

[B52] Frankowski J. C. DeMars K. M. Ahmad A. S. Hawkins K. E. Yang C. Leclerc J. L. (2015). Detrimental role of the EP1 prostanoid receptor in blood-brain barrier damage following experimental ischemic stroke. *Sci. Rep.* 5:17956. 10.1038/srep17956 PMC4673693 26648273

[B53] Friedman A. (2011). Blood-brain barrier dysfunction, status epilepticus, seizures, and epilepsy: a puzzle of a chicken and egg? *Epilepsia* 52(Suppl 8) 19–20. 10.1111/j.1528-1167.2011.03227.x 21967353 PMC3234990

[B54] Fukuhara S. Sakurai A. Sano H. Yamagishi A. Somekawa S. Takakura N. (2005). Cyclic AMP potentiates vascular endothelial cadherin-mediated cell-cell contact to enhance endothelial barrier function through an Epac-Rap1 signaling pathway. *Mol. Cell. Biol.* 25 136–146. 10.1128/mcb.25.1.136-146.2005 15601837 PMC538793

[B55] Fukumoto K. Takagi N. Yamamoto R. Moriyama Y. Takeo S. Tanonaka K. (2010). Prostanoid EP1 receptor antagonist reduces blood-brain barrier leakage after cerebral ischemia. *Eur. J. Pharmacol.* 640 82–86. 10.1016/j.ejphar.2010.05.001 20470769

[B56] Garbuzova-Davis S. Hernandez-Ontiveros D. G. Rodrigues M. C. Haller E. Frisina-Deyo A. Mirtyl S. (2012). Impaired blood-brain/spinal cord barrier in ALS patients. *Brain Res.* 1469 114–128. 10.1016/j.brainres.2012.05.056 22750125

[B57] Ge X. Li W. Huang S. Yin Z. Yang M. Han Z. (2019). Increased miR-21-3p in injured brain microvascular endothelial cells after traumatic brain injury aggravates blood-brain barrier damage by promoting cellular apoptosis and inflammation through targeting MAT2B. *J. Neurotrauma* 36 1291–1305. 10.1089/neu.2018.5728 29695199

[B58] Gelosa P. Colazzo F. Tremoli E. Sironi L. Castiglioni L. (2017). Cysteinyl leukotrienes as potential pharmacological targets for cerebral diseases. *Mediators Inflamm.* 2017:3454212. 10.1155/2017/3454212 PMC5451784 28607533

[B59] Gray M. T. Woulfe J. M. (2015). Striatal blood-brain barrier permeability in Parkinson’s disease. *J. Cereb. Blood Flow Metab.* 35 747–750. 10.1038/jcbfm.2015.32 25757748 PMC4420870

[B60] Haj-Yasein N. N. Vindedal G. F. Eilert-Olsen M. Gundersen G. A. Skare Ø Laake P. (2011). Glial-conditional deletion of aquaporin-4 (Aqp4) reduces blood-brain water uptake and confers barrier function on perivascular astrocyte endfeet. *Proc. Natl. Acad. Sci. U.S.A.* 108 17815–17820. 10.1073/pnas.1110655108 21990350 PMC3203818

[B61] Hao F. L. Han X. F. Wang X. L. Zhao Z. R. Guo A. H. Lu X. J. (2019). The neurovascular protective effect of alogliptin in murine MCAO model and brain endothelial cells. *Biomed. Pharmacother.* 109 181–187. 10.1016/j.biopha.2018.10.064 30396075

[B62] Hao Y. Guo M. Feng Y. Dong Q. Cui M. (2020). Lysophospholipids and their g-coupled protein signaling in Alzheimer’s disease: from physiological performance to pathological impairment. *Front. Mol. Neurosci.* 13:58. 10.3389/fnmol.2020.00058 32351364 PMC7174595

[B63] Hay J. R. Johnson V. E. Young A. M. Smith D. H. Stewart W. (2015). Blood-brain barrier disruption is an early event that may persist for many years after traumatic brain injury in humans. *J. Neuropathol. Exp. Neurol.* 74 1147–1157. 10.1093/jnen/74.12.1147 26574669 PMC8744142

[B64] Horai S. Nakagawa S. Tanaka K. Morofuji Y. Couraud P. O. Deli M. A. (2013). Cilostazol strengthens barrier integrity in brain endothelial cells. *Cell Mol. Neurobiol.* 33 291–307. 10.1007/s10571-012-9896-1 23224787 PMC11497939

[B65] Hu Y. L. Wang H. Huang Q. Wang G. Zhang H. B. (2018). MicroRNA-23a-3p promotes the perihematomal edema formation after intracerebral hemorrhage via ZO-1. *Eur. Rev. Med. Pharmacol. Sci.* 22 2809–2816. 29771433 10.26355/eurrev_201805_14980

[B66] Huang L. Y. Stuart C. Takeda K. D’Agnillo F. Golding B. (2016). Poly(I:C) induces human lung endothelial barrier dysfunction by disrupting tight junction expression of claudin-5. *PLoS One* 11:e0160875. 10.1371/journal.pone.0160875 27504984 PMC4978501

[B67] Hudson S. J. Cai J. P. Thomas V. Chin Y. H. (1996). Intracellular signaling of tumor necrosis factor-alpha in brain microvascular endothelial cells is mediated by a protein tyrosine kinase and protein kinase C-dependent pathway. *J. Neuroimmunol.* 70 199–206. 10.1016/s0165-5728(96)00116-6 8898728

[B68] Igarashi Y. Utsumi H. Chiba H. Yamada-Sasamori Y. Tobioka H. Kamimura Y. (1999). Glial cell line-derived neurotrophic factor induces barrier function of endothelial cells forming the blood-brain barrier. *Biochem. Biophys. Res. Commun.* 261 108–112. 10.1006/bbrc.1999.0992 10405331

[B69] Ikeda-Matsuo Y. Tanji H. Narumiya S. Sasaki Y. (2011). Inhibition of prostaglandin E2 EP3 receptors improves stroke injury via anti-inflammatory and anti-apoptotic mechanisms. *J. Neuroimmunol.* 238 34–43. 10.1016/j.jneuroim.2011.06.014 21803432

[B70] Ishizaki T. Chiba H. Kojima T. Fujibe M. Soma T. Miyajima H. (2003). Cyclic AMP induces phosphorylation of claudin-5 immunoprecipitates and expression of claudin-5 gene in blood-brain-barrier endothelial cells via protein kinase A-dependent and -independent pathways. *Exp. Cell Res.* 290 275–288. 10.1016/s0014-4827(03)00354-9 14567987

[B71] Jaureguiberry-Bravo M. Wilson R. Carvallo L. Berman J. W. (2016). Opioids and opioid maintenance therapies: their impact on monocyte-mediated HIV neuropathogenesis. *Curr. HIV Res.* 14 417–430. 10.2174/1570162x14666160324124132 27009099 PMC5487025

[B72] Ji Y. B. Gao Q. Tan X. X. Huang X. W. Ma Y. Z. Fang C. (2021). Lithium alleviates blood-brain barrier breakdown after cerebral ischemia and reperfusion by upregulating endothelial Wnt/β-catenin signaling in mice. *Neuropharmacology* 186:108474. 10.1016/j.neuropharm.2021.108474 33524408

[B73] Johansson A. Engler H. Blomquist G. Scott B. Wall A. Aquilonius S. M. (2007). Evidence for astrocytosis in ALS demonstrated by [11C](L)-deprenyl-D2 PET. *J. Neurol. Sci.* 255 17–22. 10.1016/j.jns.2007.01.057 17346749

[B74] Ju F. Ran Y. Zhu L. Cheng X. Gao H. Xi X. (2018). Increased BBB permeability enhances activation of microglia and exacerbates loss of dendritic spines after transient global cerebral ischemia. *Front. Cell Neurosci.* 12:236. 10.3389/fncel.2018.00236 30123113 PMC6085918

[B75] Kalani A. Kamat P. K. Familtseva A. Chaturvedi P. Muradashvili N. Narayanan N. (2014). Role of microRNA29b in blood-brain barrier dysfunction during hyperhomocysteinemia: an epigenetic mechanism. *J. Cereb. Blood Flow Metab.* 34 1212–1222. 10.1038/jcbfm.2014.74 24802332 PMC4083388

[B76] Kerage D. Brindley D. N. Hemmings D. G. (2014). Review: novel insights into the regulation of vascular tone by sphingosine 1-phosphate. *Placenta* 35 S86–S92. 24411702 10.1016/j.placenta.2013.12.006

[B77] Kettenmann H. Hanisch U. K. Noda M. Verkhratsky A. (2011). Physiology of microglia. *Physiol. Rev.* 91 461–553. 21527731 10.1152/physrev.00011.2010

[B78] Kim D. G. Jang M. Choi S. H. Kim H. J. Jhun H. Kim H. C. (2018). Gintonin, a ginseng-derived exogenous lysophosphatidic acid receptor ligand, enhances blood-brain barrier permeability and brain delivery. *Int. J. Biol. Macromol.* 114 1325–1337. 10.1016/j.ijbiomac.2018.03.158 29604355

[B79] Kim G. S. Yang L. Zhang G. Zhao H. Selim M. McCullough L. D. (2015). Critical role of sphingosine-1-phosphate receptor-2 in the disruption of cerebrovascular integrity in experimental stroke. *Nat. Commun.* 6:7893. 10.1038/ncomms8893 PMC4587559 26243335

[B80] Kimura I. Dohgu S. Takata F. Matsumoto J. Watanabe T. Iwao T. (2020). Oligodendrocytes upregulate blood-brain barrier function through mechanisms other than the PDGF-BB/PDGFRα pathway in the barrier-tightening effect of oligodendrocyte progenitor cells. *Neurosci. Lett.* 715:134594. 10.1016/j.neulet.2019.134594 31678431

[B81] Krasnow S. M. Knoll J. G. Verghese S. C. Levasseur P. R. Marks D. L. (2017). Amplification and propagation of interleukin-1beta signaling by murine brain endothelial and glial cells. *J. Neuroinflammation* 14:133. 10.1186/s12974-017-0908-4 PMC5494131 28668091

[B82] Kuan W. L. Bennett N. He X. Skepper J. N. Martynyuk N. Wijeyekoon R. (2016). α-Synuclein pre-formed fibrils impair tight junction protein expression without affecting cerebral endothelial cell function. *Exp. Neurol.* 285 72–81. 10.1016/j.expneurol.2016.09.003 27632900

[B83] Laksitorini M. D. Yathindranath V. Xiong W. Hombach-Klonisch S. Miller D. W. (2019). Modulation of Wnt/beta-catenin signaling promotes blood-brain barrier phenotype in cultured brain endothelial cells. *Sci. Rep.* 9:19718. 10.1038/s41598-019-56075-w PMC6928218 31873116

[B84] Laurent C. Dorothée G. Hunot S. Martin E. Monnet Y. Duchamp M. (2017). Hippocampal T cell infiltration promotes neuroinflammation and cognitive decline in a mouse model of tauopathy. *Brain* 140 184–200. 10.1093/brain/aww270 27818384 PMC5382942

[B85] Lecuyer M. A. Kebir H. Prat A. (2016). Glial influences on BBB functions and molecular players in immune cell trafficking. *Biochim. Biophys. Acta* 1862 472–482. 10.1016/j.bbadis.2015.10.004 26454208

[B86] Lee H. J. Suk J. E. Patrick C. Bae E. J. Cho J. H. Rho S. (2010). Direct transfer of alpha-synuclein from neuron to astroglia causes inflammatory responses in synucleinopathies. *J. Biol. Chem.* 285 9262–9272. 10.1074/jbc.m109.081125 20071342 PMC2838344

[B87] Lee S. W. Kim W. J. Choi Y. K. Song H. S. Son M. J. Gelman I. H. (2003). SSeCKS regulates angiogenesis and tight junction formation in blood-brain barrier. *Nat. Med.* 9 900–906. 10.1038/nm889 12808449

[B88] Li L. Sluter M. N. Yu Y. Jiang J. (2020). Prostaglandin E receptors as targets for ischemic stroke: Novel evidence and molecular mechanisms of efficacy. *Pharmacol. Res.* 163:105238. 10.1016/j.phrs.2020.105238 33053444 PMC7854947

[B89] Liang X. Lin L. Woodling N. S. Wang Q. Anacker C. Pan T. (2011). Signaling via the prostaglandin E(2) receptor EP4 exerts neuronal and vascular protection in a mouse model of cerebral ischemia. *J. Clin. Invest.* 121 4362–4371. 10.1172/jci46279 21965326 PMC3204834

[B90] Liddelow S. A. Guttenplan K. A. Clarke L. E. Bennett F. C. Bohlen C. J. Schirmer L. (2017). Neurotoxic reactive astrocytes are induced by activated microglia. *Nature* 541 481–487. 28099414 10.1038/nature21029 PMC5404890

[B91] Lier J. Ondruschka B. Bechmann I. Dreßler J. (2020). Fast microglial activation after severe traumatic brain injuries. *Int. J. Legal Med.* 134 2187–2193. 10.1007/s00414-020-02308-x 32372233 PMC7578125

[B92] Lin M. Zhu L. Wang J. Xue Y. Shang X. (2019). miR-424-5p maybe regulate blood-brain barrier permeability in a model in vitro with Abeta incubated endothelial cells. *Biochem. Biophys. Res. Commun.* 517 525–531. 10.1016/j.bbrc.2019.07.075 31375213

[B93] Liu M. Xu Z. Wang L. Zhang L. Liu Y. Cao J. (2020). Cottonseed oil alleviates ischemic stroke injury by inhibiting the inflammatory activation of microglia and astrocyte. *J. Neuroinflammation* 17:270. 10.1186/s12974-020-01946-7 PMC7488511 32917229

[B94] Liu Q. Zhu L. Liu X. Zheng J. Liu Y. Ruan X. (2020). TRA2A-induced upregulation of LINC00662 regulates blood-brain barrier permeability by affecting ELK4 mRNA stability in Alzheimer’s microenvironment. *RNA Biol.* 17 1293–1308. 10.1080/15476286.2020.1756055 32372707 PMC7549634

[B95] Logsdon A. F. Meabon J. S. Cline M. M. Bullock K. M. Raskind M. A. Peskind E. R. (2018). Blast exposure elicits blood-brain barrier disruption and repair mediated by tight junction integrity and nitric oxide dependent processes. *Sci. Rep.* 8:11344. 10.1038/s41598-018-29341-6 PMC6063850 30054495

[B96] Lopez-Ramirez M. A. Fischer R. Torres-Badillo C. C. Davies H. A. Logan K. Pfizenmaier K. (2012). Role of caspases in cytokine-induced barrier breakdown in human brain endothelial cells. *J. Immunol.* 189 3130–3139. 10.4049/jimmunol.1103460 22896632

[B97] Lorenowicz M. J. Fernandez-Borja M. Kooistra M. R. Bos J. L. Hordijk P. L. (2008). PKA and Epac1 regulate endothelial integrity and migration through parallel and independent pathways. *Eur. J. Cell Biol.* 87 779–792. 10.1016/j.ejcb.2008.05.004 18635287

[B98] Lu L. Hogan-Cann A. D. Globa A. K. Lu P. Nagy J. I. Bamji S. X. (2019). Astrocytes drive cortical vasodilatory signaling by activating endothelial NMDA receptors. *J. Cereb. Blood Flow Metab.* 39 481–496. 29072857 10.1177/0271678X17734100 PMC6421257

[B99] Lucas R. Garcia I. Donati Y. R. Hribar M. Mandriota S. J. Giroud C. (1998). Both TNF receptors are required for direct TNF-mediated cytotoxicity in microvascular endothelial cells. *Eur. J. Immunol.* 28 3577–3586. 10.1002/(sici)1521-4141(199811)28:11<3577::aid-immu3577>3.0.co;2-# 9842900

[B100] Luissint A. C. Artus C. Glacial F. Ganeshamoorthy K. Couraud P. O. (2012). Tight junctions at the blood brain barrier: physiological architecture and disease-associated dysregulation. *Fluids Barriers CNS* 9:23. 10.1186/2045-8118-9-23 23140302 PMC3542074

[B101] Ma F. Zhang X. Yin K. J. (2020). MicroRNAs in central nervous system diseases: A prospective role in regulating blood-brain barrier integrity. *Exp. Neurol.* 323:113094. 10.1016/j.expneurol.2019.113094 31676317 PMC7304262

[B102] Machida T. Dohgu S. Takata F. Matsumoto J. Kimura I. Koga M. (2017a). Role of thrombin-PAR1-PKCθ/δ axis in brain pericytes in thrombin-induced MMP-9 production and blood-brain barrier dysfunction in vitro. *Neuroscience* 350 146–157. 10.1016/j.neuroscience.2017.03.026 28344073

[B103] Machida T. Takata F. Matsumoto J. Miyamura T. Hirata R. Kimura I. (2017b). Contribution of thrombin-reactive brain pericytes to blood-brain barrier dysfunction in an in vivo mouse model of obesity-associated diabetes and an in vitro rat model. *PLoS One* 12:e0177447. 10.1371/journal.pone.0177447 28489922 PMC5425209

[B104] Machida T. Takata F. Matsumoto J. Takenoshita H. Kimura I. Yamauchi A. (2015). Brain pericytes are the most thrombin-sensitive matrix metalloproteinase-9-releasing cell type constituting the blood-brain barrier in vitro. *Neurosci. Lett.* 599 109–114. 10.1016/j.neulet.2015.05.028 26002077

[B105] Maki T. Maeda M. Uemura M. Lo E. K. Terasaki Y. Liang A. C. (2015). Potential interactions between pericytes and oligodendrocyte precursor cells in perivascular regions of cerebral white matter. *Neurosci. Lett.* 597 164–169. 10.1016/j.neulet.2015.04.047 25936593 PMC4443478

[B106] Mark K. S. Trickler W. J. Miller D. W. (2001). Tumor necrosis factor-alpha induces cyclooxygenase-2 expression and prostaglandin release in brain microvessel endothelial cells. *J. Pharmacol. Exp. Ther.* 297 1051–1058. 11356928

[B107] Masago K. Kihara Y. Yanagida K. Hamano F. Nakagawa S. Niwa M. (2018). Lysophosphatidic acid receptor, LPA6, regulates endothelial blood-brain barrier function: Implication for hepatic encephalopathy. *Biochem. Biophys. Res. Commun.* 501 1048–1054. 10.1016/j.bbrc.2018.05.106 29778535 PMC6108890

[B108] Matsumoto J. Dohgu S. Takata F. Iwao T. Kimura I. Tomohiro M. (2020). Serum amyloid A-induced blood-brain barrier dysfunction associated with decreased claudin-5 expression in rat brain endothelial cells and its inhibition by high-density lipoprotein in vitro. *Neurosci. Lett.* 738:135352. 10.1016/j.neulet.2020.135352 32931862

[B109] Matsumoto J. Dohgu S. Takata F. Machida T. Bölükbaşi Hatip F. F. Hatip-Al-Khatib I. (2018). TNF-α-sensitive brain pericytes activate microglia by releasing IL-6 through cooperation between IκB-NFκB and JAK-STAT3 pathways. *Brain Res.* 1692 34–44. 10.1016/j.brainres.2018.04.023 29702085

[B110] Matsumoto J. Dohgu S. Takata F. Nishioku T. Sumi N. Machida T. (2012). Lipopolysaccharide-activated microglia lower P-glycoprotein function in brain microvascular endothelial cells. *Neurosci. Lett.* 524 45–48. 10.1016/j.neulet.2012.07.004 22801252

[B111] Matsumoto J. Takata F. Machida T. Takahashi H. Soejima Y. Funakoshi M. (2014). Tumor necrosis factor-α-stimulated brain pericytes possess a unique cytokine and chemokine release profile and enhance microglial activation. *Neurosci. Lett.* 578 133–138. 10.1016/j.neulet.2014.06.052 24993300

[B112] Mayne K. White J. A. McMurran C. E. Rivera F. J. de la Fuente A. G. (2020). Aging and neurodegenerative disease: is the adaptive immune system a friend or foe? *Front. Aging Neurosci.* 12:572090. 10.3389/fnagi.2020.572090 33173502 PMC7538701

[B113] Megra B. W. Eugenin E. A. Berman J. W. (2018). Inflammatory mediators reduce surface PrP(c) on human BMVEC resulting in decreased barrier integrity. *Lab. Invest.* 98 1347–1359. 10.1038/s41374-018-0090-z 29959417 PMC6163073

[B114] Merlini M. Rafalski V. A. Rios Coronado P. E. Gill T. M. Ellisman M. Muthukumar G. (2019). Fibrinogen induces microglia-mediated spine elimination and cognitive impairment in an Alzheimer’s disease model. *Neuron* 101 1099–1108.e6. 30737131 10.1016/j.neuron.2019.01.014 PMC6602536

[B115] Michinaga S. Koyama Y. (2019). Dual roles of astrocyte-derived factors in regulation of blood-brain barrier function after brain damage. *Int. J. Mol. Sci.* 20:571. 10.3390/ijms20030571 30699952 PMC6387062

[B116] Miller F. Fenart L. Landry V. Coisne C. Cecchelli R. Dehouck M. P. (2005). The MAP kinase pathway mediates transcytosis induced by TNF-alpha in an in vitro blood-brain barrier model. *Eur. J. Neurosci.* 22 835–844. 10.1111/j.1460-9568.2005.04273.x 16115207

[B117] Minagar A. Alexander J. S. (2003). Blood-brain barrier disruption in multiple sclerosis. *Mult. Scler.* 9 540–549. 10.1191/1352458503ms965oa 14664465

[B118] Mishra R. Singh S. K. (2013). HIV-1 Tat C modulates expression of miRNA-101 to suppress VE-cadherin in human brain microvascular endothelial cells. *J. Neurosci.* 33 5992–6000. 10.1523/jneurosci.4796-12.2013 23554480 PMC6618916

[B119] Miyazaki K. Ohta Y. Nagai M. Morimoto N. Kurata T. Takehisa Y. (2011). Disruption of neurovascular unit prior to motor neuron degeneration in amyotrophic lateral sclerosis. *J. Neurosci. Res.* 89 718–728. 10.1002/jnr.22594 21337372

[B120] Mohan S. Koller E. J. Fazal J. A. De Oliveria G. Pawlowicz A. I. Dore S. (2018). Genetic deletion of PGF2alpha-FP receptor exacerbates brain injury following experimental intracerebral hemorrhage. *Front. Neurosci.* 12:556. 10.3389/fnins.2018.00556 30233287 PMC6134069

[B121] Montagne A. Barnes S. R. Sweeney M. D. Halliday M. R. Sagare A. P. Zhao Z. (2015). Blood-brain barrier breakdown in the aging human hippocampus. *Neuron* 85 296–302. 10.1016/j.neuron.2014.12.032 25611508 PMC4350773

[B122] Morofuji Y. Nakagawa S. So G. Hiu T. Horai S. Hayashi K. (2010). Pitavastatin strengthens the barrier integrity in primary cultures of rat brain endothelial cells. *Cell. Mol. Neurobiol.* 30 727–735. 10.1007/s10571-010-9497-9 20127168 PMC11498758

[B123] Murakami M. (2011). Lipid mediators in life science. *Exp. Anim.* 60 7–20. 10.1538/expanim.60.7 21325748

[B124] Murakami M. Nguyen L. T. Zhuang Z. W. Moodie K. L. Carmeliet P. Stan R. V. (2008). The FGF system has a key role in regulating vascular integrity. *J. Clin. Invest.* 118 3355–3366. 10.1172/jci35298 18776942 PMC2528913

[B125] Muramatsu R. Kuroda M. Matoba K. Lin H. Takahashi C. Koyama Y. (2015). Prostacyclin prevents pericyte loss and demyelination induced by lysophosphatidylcholine in the central nervous system. *J. Biol. Chem.* 290 11515–11525. 10.1074/jbc.m114.587253 25795781 PMC4416855

[B126] Nagib M. M. Yu Y. Jiang J. (2020). Targeting prostaglandin receptor EP2 for adjunctive treatment of status epilepticus. *Pharmacol. Ther.* 209:107504. 10.1016/j.pharmthera.2020.107504 32088247 PMC7222917

[B127] Nakagawa S. Aruga J. (2020). Sphingosine 1-phosphate signaling is involved in impaired blood-brain barrier function in ischemia-reperfusion injury. *Mol. Neurobiol.* 57 1594–1606. 10.1007/s12035-019-01844-x 31802363

[B128] Nakagawa S. Deli M. A. Kawaguchi H. Shimizudani T. Shimono T. Kittel A. (2009). A new blood-brain barrier model using primary rat brain endothelial cells, pericytes and astrocytes. *Neurochem. Int.* 54 253–263. 10.1016/j.neuint.2008.12.002 19111869

[B129] Navarro R. Compte M. Alvarez-Vallina L. Sanz L. (2016). Immune regulation by pericytes: modulating innate and adaptive immunity. *Front. Immunol.* 7:480. 10.3389/fimmu.2016.00480 PMC5095456 27867386

[B130] Nelson A. R. Sweeney M. D. Sagare A. P. Zlokovic B. V. (2016). Neurovascular dysfunction and neurodegeneration in dementia and Alzheimer’s disease. *Biochim. Biophys. Acta* 1862 887–900. 10.1016/j.bbadis.2015.12.016 26705676 PMC4821735

[B131] Ni Y. Teng T. Li R. Simonyi A. Sun G. Y. Lee J. C. (2017). TNFalpha alters occludin and cerebral endothelial permeability: role of p38MAPK. *PLoS One* 12:e0170346. 10.1371/journal.pone.0170346 28170408 PMC5295672

[B132] Nishihara H. Shimizu F. Kitagawa T. Yamanaka N. Akada J. Kuramitsu Y. (2017). Identification of galectin-3 as a possible antibody target for secondary progressive multiple sclerosis. *Mult. Scler.* 23 382–394. 10.1177/1352458516655217 27339072

[B133] Nishioku T. Dohgu S. Takata F. Eto T. Ishikawa N. Kodama K. B. (2009). Detachment of brain pericytes from the basal lamina is involved in disruption of the blood-brain barrier caused by lipopolysaccharide-induced sepsis in mice. *Cell. Mol. Neurobiol.* 29 309–316. 10.1007/s10571-008-9322-x 18987969 PMC11506181

[B134] Nishioku T. Matsumoto J. Dohgu S. Sumi N. Miyao K. Takata F. (2010). Tumor necrosis factor-alpha mediates the blood-brain barrier dysfunction induced by activated microglia in mouse brain microvascular endothelial cells. *J. Pharmacol. Sci.* 112 251–254. 10.1254/jphs.09292sc 20118615

[B135] O’Carroll S. J. Kho D. T. Wiltshire R. Nelson V. Rotimi O. Johnson R. (2015). Pro-inflammatory TNFα and IL-1β differentially regulate the inflammatory phenotype of brain microvascular endothelial cells. *J. Neuroinflammation* 12:131. 10.1186/s12974-015-0346-0 PMC4506411 26152369

[B136] O’Keeffe E. Kelly E. Liu Y. Giordano C. Wallace E. Hynes M. (2020). Dynamic blood-brain barrier regulation in mild traumatic brain injury. *J. Neurotrauma* 37 347–356. 31702476 10.1089/neu.2019.6483 PMC10331162

[B137] Oliva A. A. Jr. Kang Y. Sanchez-Molano J. Furones C. Atkins C. M. (2012). STAT3 signaling after traumatic brain injury. *J. Neurochem.* 120 710–720. 10.1111/j.1471-4159.2011.07610.x 22145815

[B138] Pan W. Kastin A. J. (2002). TNFalpha transport across the blood-brain barrier is abolished in receptor knockout mice. *Exp. Neurol.* 174 193–200. 10.1006/exnr.2002.7871 11922661

[B139] Paolinelli R. Corada M. Ferrarini L. Devraj K. Artus C. Czupalla C. J. (2013). Wnt activation of immortalized brain endothelial cells as a tool for generating a standardized model of the blood brain barrier in vitro. *PLoS One* 8:e70233. 10.1371/journal.pone.0070233 23940549 PMC3734070

[B140] Peng J. He F. Zhang C. Deng X. Yin F. (2011). Protein kinase C-α signals P115RhoGEF phosphorylation and RhoA activation in TNF-α-induced mouse brain microvascular endothelial cell barrier dysfunction. *J. Neuroinflammation* 8:28. 10.1186/1742-2094-8-28 21473788 PMC3080812

[B141] Perrot C. Y. Herrera J. L. Fournier-Goss A. E. Komatsu M. (2020). Prostaglandin E2 breaks down pericyte-endothelial cell interaction via EP1 and EP4-dependent downregulation of pericyte N-cadherin, connexin-43, and R-Ras. *Sci. Rep.* 10:11186. 10.1038/s41598-020-68019-w PMC7341885 32636414

[B142] Petersen M. A. Ryu J. K. Akassoglou K. (2018). Fibrinogen in neurological diseases: mechanisms, imaging and therapeutics. *Nat. Rev. Neurosci.* 19 283–301. 10.1038/nrn.2018.13 29618808 PMC6743980

[B143] Pham L. D. Hayakawa K. Seo J. H. Nguyen M. N. Som A. T. Lee B. J. (2012). Crosstalk between oligodendrocytes and cerebral endothelium contributes to vascular remodeling after white matter injury. *Glia* 60 875–881. 10.1002/glia.22320 22392631 PMC3325331

[B144] Pieper C. Pieloch P. Galla H. J. (2013). Pericytes support neutrophil transmigration via interleukin-8 across a porcine co-culture model of the blood-brain barrier. *Brain Res.* 1524 1–11. 10.1016/j.brainres.2013.05.047 23769734

[B145] Pluimer B. R. Colt M. Zhao Z. (2020). G protein-coupled receptors in the mammalian blood-brain barrier. *Front. Cell Neurosci.* 14:139. 10.3389/fncel.2020.00139 PMC7283493 32581715

[B146] Prasain N. Alexeyev M. Balczon R. Stevens T. (2009). Soluble adenylyl cyclase-dependent microtubule disassembly reveals a novel mechanism of endothelial cell retraction. *Am. J. Physiol. Lung Cell. Mol. Physiol.* 297 L73–L83. 19395666 10.1152/ajplung.90577.2008 PMC2711814

[B147] Quinn J. J. Chang H. Y. (2016). Unique features of long non-coding RNA biogenesis and function. *Nat. Rev. Genet.* 17 47–62. 10.1038/nrg.2015.10 26666209

[B148] Ralay Ranaivo H. Hodge J. N. Choi N. Wainwright M. S. (2012). Albumin induces upregulation of matrix metalloproteinase-9 in astrocytes via MAPK and reactive oxygen species-dependent pathways. *J. Neuroinflammation* 9:68. 10.1186/1742-2094-9-68 PMC3419618 22507553

[B149] Rigor R. R. Beard R. S. Jr. Litovka O. P. Yuan S. Y. (2012). Interleukin-1beta-induced barrier dysfunction is signaled through PKC-theta in human brain microvascular endothelium. *Am. J. Physiol. Cell. Physiol.* 302 C1513–C1522. 22403784 10.1152/ajpcell.00371.2011 PMC3362001

[B150] Rochfort K. D. Collins L. E. McLoughlin A. Cummins P. M. (2016). Tumour necrosis factor-alpha-mediated disruption of cerebrovascular endothelial barrier integrity in vitro involves the production of proinflammatory interleukin-6. *J. Neurochem.* 136 564–572. 10.1111/jnc.13408 26499872

[B151] Rochfort K. D. Collins L. E. Murphy R. P. Cummins P. M. (2014). Downregulation of blood-brain barrier phenotype by proinflammatory cytokines involves NADPH oxidase-dependent ROS generation: consequences for interendothelial adherens and tight junctions. *PLoS One* 9:e101815. 10.1371/journal.pone.0101815 24992685 PMC4081725

[B152] Rochfort K. D. Cummins P. M. (2015). Cytokine-mediated dysregulation of zonula occludens-1 properties in human brain microvascular endothelium. *Microvasc. Res.* 100 48–53. 10.1016/j.mvr.2015.04.010 25953589

[B153] Rojas A. Chen D. Ganesh T. Varvel N. H. Dingledine R. (2019). The COX-2/prostanoid signaling cascades in seizure disorders. *Expert Opin. Ther. Targets* 23 1–13. 10.1080/14728222.2019.1554056 30484341 PMC6481174

[B154] Rubin L. L. Hall D. E. Porter S. Barbu K. Cannon C. Horner H. C. (1991). A cell culture model of the blood-brain barrier. *J. Cell Biol.* 115 1725–1735. 1661734 10.1083/jcb.115.6.1725 PMC2289219

[B155] Rudziak P. Ellis C. G. Kowalewska P. M. (2019). Role and molecular mechanisms of pericytes in regulation of leukocyte diapedesis in inflamed tissues. *Mediators Inflamm.* 2019:4123605. 10.1155/2019/4123605 PMC6530229 31205449

[B156] Ruprecht K. Kuhlmann T. Seif F. Hummel V. Kruse N. Brück W. (2001). Effects of oncostatin M on human cerebral endothelial cells and expression in inflammatory brain lesions. *J. Neuropathol. Exp. Neurol.* 60 1087–1098. 10.1093/jnen/60.11.1087 11706938

[B157] Sack G. H. Jr. (2018). Serum amyloid A–a review. *Mol. Med.* 24:46. 10.1186/s10020-018-0047-0 PMC6117975 30165816

[B158] Sakai K. Takata F. Yamanaka G. Yasunaga M. Hashiguchi K. Tominaga K. (2021). Reactive pericytes in early phase are involved in glial activation and late-onset hypersusceptibility to pilocarpine-induced seizures in traumatic brain injury model mice. *J. Pharmacol. Sci.* 145 155–165. 10.1016/j.jphs.2020.11.008 33357774

[B159] Sayner S. L. (2011). Emerging themes of cAMP regulation of the pulmonary endothelial barrier. *Am. J. Physiol. Lung Cell. Mol. Physiol.* 300 L667–L678. 21335524 10.1152/ajplung.00433.2010 PMC4631543

[B160] Sayner S. L. Frank D. W. King J. Chen H. VandeWaa J. Stevens T. (2004). Paradoxical cAMP-induced lung endothelial hyperpermeability revealed by *Pseudomonas aeruginosa* ExoY. *Circ. Res.* 95 196–203. 10.1161/01.res.0000134922.25721.d9 15192021

[B161] Schoknecht K. Prager O. Vazana U. Kamintsky L. Harhausen D. Zille M. (2014). Monitoring stroke progression: in vivo imaging of cortical perfusion, blood-brain barrier permeability and cellular damage in the rat photothrombosis model. *J. Cereb. Blood Flow Metab.* 34 1791–1801. 10.1038/jcbfm.2014.147 25160672 PMC4269756

[B162] Schreibelt G. Kooij G. Reijerkerk A. van Doorn R. Gringhuis S. I. van der Pol S. (2007). Reactive oxygen species alter brain endothelial tight junction dynamics via RhoA, PI3 kinase, and PKB signaling. *FASEB J.* 21 3666–3676. 10.1096/fj.07-8329com 17586731

[B163] Seo J. H. Maki T. Maeda M. Miyamoto N. Liang A. C. Hayakawa K. (2014). Oligodendrocyte precursor cells support blood-brain barrier integrity via TGF-β signaling. *PLoS One* 9:e103174. 10.1371/journal.pone.0103174 25078775 PMC4117639

[B164] Seo J. H. Miyamoto N. Hayakawa K. Pham L. D. Maki T. Ayata C. (2013). Oligodendrocyte precursors induce early blood-brain barrier opening after white matter injury. *J. Clin. Invest.* 123 782–786. 23281396 10.1172/JCI65863 PMC3561802

[B165] Setiadi H. Yago T. Liu Z. McEver R. P. (2019). Endothelial signaling by neutrophil-released oncostatin M enhances P-selectin-dependent inflammation and thrombosis. *Blood Adv.* 3 168–183. 10.1182/bloodadvances.2018026294 30670533 PMC6341191

[B166] Shan Y. Tan S. Lin Y. Liao S. Zhang B. Chen X. (2019). The glucagon-like peptide-1 receptor agonist reduces inflammation and blood-brain barrier breakdown in an astrocyte-dependent manner in experimental stroke. *J. Neuroinflammation* 16:242. 10.1186/s12974-019-1638-6 PMC6883580 31779652

[B167] Shang J. Yamashita T. Fukui Y. Song D. Li X. Zhai Y. (2018). Different associations of plasma biomarkers in Alzheimer’s disease, mild cognitive impairment, vascular dementia, and ischemic stroke. *J. Clin. Neurol.* 14 29–34. 10.3988/jcn.2018.14.1.29 29629537 PMC5765253

[B168] Shigemoto-Mogami Y. Hoshikawa K. Sato K. (2018). Activated microglia disrupt the blood-brain barrier and induce chemokines and cytokines in a rat in vitro model. *Front. Cell Neurosci.* 12:494. 10.3389/fncel.2018.00494 30618641 PMC6300509

[B169] Shimizu F. Nishihara H. Kanda T. (2018). Blood-brain barrier dysfunction in immuno-mediated neurological diseases. *Immunol. Med.* 41 120–128. 10.1080/25785826.2018.1531190 30938273

[B170] Shimizu F. Schaller K. L. Owens G. P. Cotleur A. C. Kellner D. Takeshita Y. (2017). Glucose-regulated protein 78 autoantibody associates with blood-brain barrier disruption in neuromyelitis optica. *Sci. Transl. Med.* 9:eaai9111. 10.1126/scitranslmed.aai9111 28679661 PMC5585784

[B171] Shimizu F. Takeshita Y. Sano Y. Hamamoto Y. Shiraishi H. Sato T. (2019). GRP78 antibodies damage the blood-brain barrier and relate to cerebellar degeneration in Lambert-Eaton myasthenic syndrome. *Brain* 142 2253–2264. 10.1093/brain/awz168 31236596

[B172] Shimizu F. Tasaki A. Sano Y. Ju M. Nishihara H. Oishi M. (2014). Sera from remitting and secondary progressive multiple sclerosis patients disrupt the blood-brain barrier. *PLoS One* 9:e92872. 10.1371/journal.pone.0092872 24686948 PMC3970956

[B173] Shimizu T. (2009). Lipid mediators in health and disease: enzymes and receptors as therapeutic targets for the regulation of immunity and inflammation. *Annu. Rev. Pharmacol. Toxicol.* 49 123–150. 10.1146/annurev.pharmtox.011008.145616 18834304

[B174] Skinner R. A. Gibson R. M. Rothwell N. J. Pinteaux E. Penny J. I. (2009). Transport of interleukin-1 across cerebromicrovascular endothelial cells. *Br. J. Pharmacol.* 156 1115–1123. 10.1111/j.1476-5381.2008.00129.x 19298391 PMC2697691

[B175] So G. Nakagawa S. Morofuji Y. Hiu T. Hayashi K. Tanaka K. (2015). Candesartan improves ischemia-induced impairment of the blood-brain barrier in vitro. *Cell. Mol. Neurobiol.* 35 563–572. 10.1007/s10571-014-0152-8 25547389 PMC11486288

[B176] Spampinato S. F. Merlo S. Fagone E. Fruciano M. Barbagallo C. Kanda T. (2019). Astrocytes modify migration of PBMCs Induced by β-Amyloid in a blood-brain barrier in vitro model. *Front. Cell Neurosci.* 13:337. 10.3389/fncel.2019.00337 31396056 PMC6664149

[B177] Spampinato S. F. Merlo S. Sano Y. Kanda T. Sortino M. A. (2021). Protective effect of the sphingosine-1 phosphate receptor agonist siponimod on disrupted blood brain barrier function. *Biochem. Pharmacol.* 186:114465. 10.1016/j.bcp.2021.114465 33577891

[B178] Stokum J. A. Gerzanich V. Simard J. M. (2016). Molecular pathophysiology of cerebral edema. *J. Cereb. Blood Flow Metab.* 36 513–538. 10.1177/0271678x15617172 26661240 PMC4776312

[B179] Strbian D. Durukan A. Pitkonen M. Marinkovic I. Tatlisumak E. Pedrono E. (2008). The blood-brain barrier is continuously open for several weeks following transient focal cerebral ischemia. *Neuroscience* 153 175–181. 10.1016/j.neuroscience.2008.02.012 18367342

[B180] Su X. Maguire-Zeiss K. A. Giuliano R. Prifti L. Venkatesh K. Federoff H. J. (2008). Synuclein activates microglia in a model of Parkinson’s disease. *Neurobiol. Aging* 29 1690–1701. 10.1016/j.neurobiolaging.2007.04.006 17537546 PMC2621109

[B181] Subhramanyam C. S. Wang C. Hu Q. Dheen S. T. (2019). Microglia-mediated neuroinflammation in neurodegenerative diseases. *Semin. Cell Dev. Biol.* 94 112–120. 10.1016/j.semcdb.2019.05.004 31077796

[B182] Sumi N. Nishioku T. Takata F. Matsumoto J. Watanabe T. Shuto H. (2010). Lipopolysaccharide-activated microglia induce dysfunction of the blood-brain barrier in rat microvascular endothelial cells co-cultured with microglia. *Cell. Mol. Neurobiol.* 30 247–253. 10.1007/s10571-009-9446-7 19728078 PMC11498813

[B183] Sun X. Yin Y. Kong L. Chen W. Miao C. Chen J. (2019). The effect of propofol on hypoxia-modulated expression of heat shock proteins: potential mechanism in modulating blood-brain barrier permeability. *Mol. Cell. Biochem.* 462 85–96. 10.1007/s11010-019-03612-w 31446614

[B184] Sweeney M. D. Kisler K. Montagne A. Toga A. W. Zlokovic B. V. (2018). The role of brain vasculature in neurodegenerative disorders. *Nat. Neurosci.* 21 1318–1331. 10.1038/s41593-018-0234-x 30250261 PMC6198802

[B185] Sweeney M. D. Zhao Z. Montagne A. Nelson A. R. Zlokovic B. V. (2019). Blood-brain barrier: from physiology to disease and back. *Physiol. Rev.* 99 21–78. 30280653 10.1152/physrev.00050.2017 PMC6335099

[B186] Takata F. Dohgu S. Matsumoto J. Machida T. Kaneshima S. Matsuo M. (2013). Metformin induces up-regulation of blood-brain barrier functions by activating AMP-activated protein kinase in rat brain microvascular endothelial cells. *Biochem. Biophys. Res. Commun.* 433 586–590. 10.1016/j.bbrc.2013.03.036 23523792

[B187] Takata F. Dohgu S. Matsumoto J. Machida T. Sakaguchi S. Kimura I. (2018). Oncostatin M-induced blood-brain barrier impairment is due to prolonged activation of STAT3 signaling in vitro. *J. Cell Biochem.* 119 9055–9063. 10.1002/jcb.27162 30076740

[B188] Takata F. Dohgu S. Matsumoto J. Takahashi H. Machida T. Wakigawa T. (2011). Brain pericytes among cells constituting the blood-brain barrier are highly sensitive to tumor necrosis factor-α, releasing matrix metalloproteinase-9 and migrating in vitro. *J. Neuroinflammation* 8:106. 10.1186/1742-2094-8-106 21867555 PMC3182916

[B189] Takata F. Dohgu S. Sakaguchi S. Sakai K. Yamanaka G. Iwao T. (2019). Oncostatin-M-reactive pericytes aggravate blood-brain barrier dysfunction by activating JAK/STAT3 signaling in vitro. *Neuroscience* 422 12–20. 10.1016/j.neuroscience.2019.10.014 31705893

[B190] Takata F. Sumi N. Nishioku T. Harada E. Wakigawa T. Shuto H. (2008). Oncostatin M induces functional and structural impairment of blood-brain barriers comprised of rat brain capillary endothelial cells. *Neurosci. Lett.* 441 163–166. 10.1016/j.neulet.2008.06.030 18603369

[B191] Takeshita T. Nakagawa S. Tatsumi R. So G. Hayashi K. Tanaka K. (2014). Cilostazol attenuates ischemia-reperfusion-induced blood-brain barrier dysfunction enhanced by advanced glycation endproducts via transforming growth factor-β1 signaling. *Mol. Cell. Neurosci.* 60 1–9. 10.1016/j.mcn.2014.01.006 24472843

[B192] Takeshita Y. Obermeier B. Cotleur A. C. Spampinato S. F. Shimizu F. Yamamoto E. (2017). Effects of neuromyelitis optica-IgG at the blood-brain barrier in vitro. *Neurol. Neuroimmunol. Neuroinflamm.* 4:e311. 10.1212/nxi.0000000000000311 28018943 PMC5173350

[B193] Tang H. B. Jiang X. J. Wang C. Liu S. C. (2018). S1P/S1PR3 signaling mediated proliferation of pericytes via Ras/pERK pathway and CAY10444 had beneficial effects on spinal cord injury. *Biochem. Biophys. Res. Commun.* 498 830–836. 10.1016/j.bbrc.2018.03.065 29534963

[B194] Tofts P. S. Kermode A. G. (1991). Measurement of the blood-brain barrier permeability and leakage space using dynamic MR imaging. 1. Fundamental concepts. *Magn. Reson. Med.* 17 357–367. 10.1002/mrm.1910170208 2062210

[B195] Tomkins O. Shelef I. Kaizerman I. Eliushin A. Afawi Z. Misk A. (2008). Blood-brain barrier disruption in post-traumatic epilepsy. *J. Neurol. Neurosurg. Psychiatry* 79 774–777. 17991703 10.1136/jnnp.2007.126425

[B196] Toyama K. Spin J. M. Deng A. C. Huang T. T. Wei K. Wagenhäuser M. U. (2018). MicroRNA-mediated therapy modulating blood-brain barrier disruption improves vascular cognitive impairment. *Arterioscler. Thromb. Vasc. Biol.* 38 1392–1406. 10.1161/atvbaha.118.310822 29650692

[B197] Tsai P. S. Kaufhold J. P. Blinder P. Friedman B. Drew P. J. Karten H. J. (2009). Correlations of neuronal and microvascular densities in murine cortex revealed by direct counting and colocalization of nuclei and vessels. *J. Neurosci.* 29 14553–14570. 10.1523/jneurosci.3287-09.2009 19923289 PMC4972024

[B198] Turner M. R. Cagnin A. Turkheimer F. E. Miller C. C. Shaw C. E. Brooks D. J. (2004). Evidence of widespread cerebral microglial activation in amyotrophic lateral sclerosis: an [11C](R)-PK11195 positron emission tomography study. *Neurobiol. Dis.* 15 601–609. 10.1016/j.nbd.2003.12.012 15056468

[B199] van Doorn R. Lopes Pinheiro M. A. Kooij G. Lakeman K. van het Hof B. van der Pol S. M. (2012). Sphingosine 1-phosphate receptor 5 mediates the immune quiescence of the human brain endothelial barrier. *J. Neuroinflammation* 9:133. 10.1186/1742-2094-9-133 PMC3425155 22715976

[B200] Vazana U. Veksler R. Pell G. S. Prager O. Fassler M. Chassidim Y. (2016). Glutamate-mediated blood-brain barrier opening: implications for neuroprotection and drug delivery. *J. Neurosci.* 36 7727–7739. 10.1523/jneurosci.0587-16.2016 27445149 PMC4951577

[B201] Voirin A. C. Perek N. Roche F. (2020). Inflammatory stress induced by a combination of cytokines (IL-6, IL-17, TNF-alpha) leads to a loss of integrity on bEnd.3 endothelial cells in vitro BBB model. *Brain Res.* 1730:146647. 10.1016/j.brainres.2020.146647 31911168

[B202] Vos C. M. Geurts J. J. Montagne L. van Haastert E. S. Bö L. van der Valk P. (2005). Blood-brain barrier alterations in both focal and diffuse abnormalities on postmortem MRI in multiple sclerosis. *Neurobiol. Dis.* 20 953–960. 10.1016/j.nbd.2005.06.012 16039866

[B203] Wan Y. Jin H. J. Zhu Y. Y. Fang Z. Mao L. He Q. (2018). MicroRNA-149-5p regulates blood-brain barrier permeability after transient middle cerebral artery occlusion in rats by targeting S1PR2 of pericytes. *FASEB J.* 32 3133–3148. 10.1096/fj.201701121r 29401609

[B204] Wang L. Geng J. Qu M. Yuan F. Wang Y. Pan J. (2020). Oligodendrocyte precursor cells transplantation protects blood-brain barrier in a mouse model of brain ischemia via Wnt/β-catenin signaling. *Cell Death Dis.* 11:9. 10.1038/s41419-019-2206-9 PMC6944692 31907363

[B205] Wang P. Pan R. Weaver J. Jia M. Yang X. Yang T. (2021). MicroRNA-30a regulates acute cerebral ischemia-induced blood-brain barrier damage through ZnT4/zinc pathway. *J. Cereb. Blood Flow Metab.* 41 641–655. 10.1177/0271678x20926787 32501158 PMC7922758

[B206] Wang X. Xue G. X. Liu W. C. Shu H. Wang M. Sun Y. (2017). Melatonin alleviates lipopolysaccharide-compromised integrity of blood-brain barrier through activating AMP-activated protein kinase in old mice. *Aging Cell* 16 414–421. 10.1111/acel.12572 28156052 PMC5334533

[B207] Wang Y. Wang M. D. Xia Y. P. Gao Y. Zhu Y. Y. Chen S. C. (2018). MicroRNA-130a regulates cerebral ischemia-induced blood-brain barrier permeability by targeting Homeobox A5. *FASEB J.* 32 935–944. 10.1096/fj.201700139rrr 29070584

[B208] Waubant E. (2006). Biomarkers indicative of blood-brain barrier disruption in multiple sclerosis. *Dis. Markers* 22 235–244. 10.1155/2006/709869 17124345 PMC3850823

[B209] Weiss J. M. Downie S. A. Lyman W. D. Berman J. W. (1998). Astrocyte-derived monocyte-chemoattractant protein-1 directs the transmigration of leukocytes across a model of the human blood-brain barrier. *J. Immunol.* 161 6896–6903. 9862722

[B210] Werry E. L. Bright F. M. Piguet O. Ittner L. M. Halliday G. M. Hodges J. R. (2019). Recent developments in TSPO PET imaging as a biomarker of neuroinflammation in neurodegenerative disorders. *Int. J. Mol. Sci.* 20:3161. 10.3390/ijms20133161 31261683 PMC6650818

[B211] Wiltshire R. Nelson V. Kho D. T. Angel C. E. O’Carroll S. J. Graham E. S. (2016). Regulation of human cerebro-microvascular endothelial baso-lateral adhesion and barrier function by S1P through dual involvement of S1P1 and S1P2 receptors. *Sci. Rep.* 6:19814. 10.1038/srep19814 PMC4728386 26813587

[B212] Won C. Lin Z. Kumar T. P. Li S. Ding L. Elkhal A. (2013). Autonomous vascular networks synchronize GABA neuron migration in the embryonic forebrain. *Nat. Commun.* 4:2149. 10.1038/ncomms3149 PMC3763945 23857367

[B213] Wong D. Dorovini-Zis K. Vincent S. R. (2004). Cytokines, nitric oxide, and cGMP modulate the permeability of an in vitro model of the human blood-brain barrier. *Exp. Neurol.* 190 446–455. 10.1016/j.expneurol.2004.08.008 15530883

[B214] Woodling N. S. Andreasson K. I. (2016). Untangling the web: toxic and protective effects of neuroinflammation and PGE2 signaling in Alzheimer’s disease. *ACS Chem. Neurosci.* 7 454–463. 10.1021/acschemneuro.6b00016 26979823 PMC5239037

[B215] Wu L. Ye Z. Pan Y. Li X. Fu X. Zhang B. (2018). Vascular endothelial growth factor aggravates cerebral ischemia and reperfusion-induced blood-brain-barrier disruption through regulating LOC102640519/HOXC13/ZO-1 signaling. *Exp. Cell Res.* 369 275–283. 10.1016/j.yexcr.2018.05.029 29842876

[B216] Wu Y. Wu H. Zeng J. Pluimer B. Dong S. Xie X. (2021). Mild traumatic brain injury induces microvascular injury and accelerates Alzheimer-like pathogenesis in mice. *Acta Neuropathol. Commun.* 9:74. 10.1186/s40478-021-01178-7 PMC8063402 33892818

[B217] Xing C. Arai K. Lo E. H. Hommel M. (2012). Pathophysiologic cascades in ischemic stroke. *Int. J. Stroke* 7 378–385. 10.1111/j.1747-4949.2012.00839.x 22712739 PMC3985770

[B218] Yamamoto M. Ramirez S. H. Sato S. Kiyota T. Cerny R. L. Kaibuchi K. (2008). Phosphorylation of claudin-5 and occludin by rho kinase in brain endothelial cells. *Am. J. Pathol.* 172 521–533. 10.2353/ajpath.2008.070076 18187566 PMC2312373

[B219] Yan A. Zhang T. Yang X. Shao J. Fu N. Shen F. (2016). Thromboxane A2 receptor antagonist SQ29548 reduces ischemic stroke-induced microglia/macrophages activation and enrichment, and ameliorates brain injury. *Sci. Rep.* 6:35885. 10.1038/srep35885 PMC5075919 27775054

[B220] Yanagida K. Liu C. H. Faraco G. Galvani S. Smith H. K. Burg N. (2017). Size-selective opening of the blood-brain barrier by targeting endothelial sphingosine 1-phosphate receptor 1. *Proc. Natl. Acad. Sci. U.S.A.* 114 4531–4536. 10.1073/pnas.1618659114 28396408 PMC5410849

[B221] Yang F. Zhou L. Wang D. Wang Z. Huang Q. Y. (2015). Minocycline ameliorates hypoxia-induced blood-brain barrier damage by inhibition of HIF-1α through SIRT-3/PHD-2 degradation pathway. *Neuroscience* 304 250–259. 10.1016/j.neuroscience.2015.07.051 26211444

[B222] Ye R. D. Sun L. (2015). Emerging functions of serum amyloid A in inflammation. *J. Leukoc. Biol.* 98 923–929. 10.1189/jlb.3vmr0315-080r 26130702 PMC6608020

[B223] Yin K. J. Hamblin M. Chen Y. E. (2014). Non-coding RNAs in cerebral endothelial pathophysiology: emerging roles in stroke. *Neurochem. Int.* 77 9–16. 10.1016/j.neuint.2014.03.013 24704794 PMC4177278

[B224] Yu N. Zhang S. Lu J. Li Y. Yi X. Tang L. (2017). Serum amyloid A, an acute phase protein, stimulates proliferative and proinflammatory responses of keratinocytes. *Cell Prolif.* 50:e12320. 10.1111/cpr.12320 27910163 PMC6529107

[B225] Zehendner C. M. Sebastiani A. Hugonnet A. Bischoff F. Luhmann H. J. Thal S. C. (2015). Traumatic brain injury results in rapid pericyte loss followed by reactive pericytosis in the cerebral cortex. *Sci. Rep.* 5:13497. 10.1038/srep13497 PMC4558600 26333872

[B226] Zeng C. Wang D. Chen C. Chen L. Chen B. Li L. (2020). Zafirlukast protects blood-brain barrier integrity from ischemic brain injury. *Chem. Biol. Interact.* 316:108915. 10.1016/j.cbi.2019.108915 31816286

[B227] Zhang H. Zhang S. Zhang J. Liu D. Wei J. Fang W. (2018). ZO-1 expression is suppressed by GM-CSF via miR-96/ERG in brain microvascular endothelial cells. *J. Cereb. Blood Flow Metab.* 38 809–822. 10.1177/0271678x17702668 28430012 PMC5987931

[B228] Zhang J. Dong B. Hao J. Yi S. Cai W. Luo Z. (2019). LncRNA Snhg3 contributes to dysfunction of cerebral microvascular cells in intracerebral hemorrhage rats by activating the TWEAK/Fn14/STAT3 pathway. *Life Sci.* 237:116929. 10.1016/j.lfs.2019.116929 31610210

[B229] Zhang J. Yuan L. Zhang X. Hamblin M. H. Zhu T. Meng F. (2016). Altered long non-coding RNA transcriptomic profiles in brain microvascular endothelium after cerebral ischemia. *Exp. Neurol.* 277 162–170. 10.1016/j.expneurol.2015.12.014 26746985 PMC4761283

[B230] Zhang T. Tian C. Wu J. Zhang Y. Wang J. Kong Q. (2020). MicroRNA-182 exacerbates blood-brain barrier (BBB) disruption by downregulating the mTOR/FOXO1 pathway in cerebral ischemia. *FASEB J.* 34 13762–13775. 10.1096/fj.201903092r 32808351

[B231] Zhang Y. M. Zhou Y. Qiu L. B. Ding G. R. Pang X. F. (2012). Altered expression of matrix metalloproteinases and tight junction proteins in rats following PEMF-induced BBB permeability change. *Biomed. Environ. Sci.* 25 197–202. 22998827 10.3967/0895-3988.2012.02.011

[B232] Zhao Z. Hu J. Gao X. Liang H. Yu H. Liu S. (2017). Hyperglycemia via activation of thromboxane A2 receptor impairs the integrity and function of blood-brain barrier in microvascular endothelial cells. *Oncotarget* 8 30030–30038. 10.18632/oncotarget.16273 28415790 PMC5444723

[B233] Zhu L. Lin M. Ma J. Liu W. Gao L. Wei S. (2019). The role of LINC00094/miR-224-5p (miR-497-5p)/Endophilin-1 axis in memantine mediated protective effects on blood-brain barrier in AD microenvironment. *J. Cell. Mol. Med.* 23 3280–3292. 10.1111/jcmm.14214 30801976 PMC6484416

[B234] Zlokovic B. V. (2008). The blood-brain barrier in health and chronic neurodegenerative disorders. *Neuron* 57 178–201. 10.1016/j.neuron.2008.01.003 18215617

