# Supplementary material for: Blood-Brain Barrier Dysfunction Amplifies the Development of Neuroinflammation: Understanding of Cellular Events in Brain Microvascular Endothelial Cells for Prevention and Treatment of BBB Dysfunction
Source: Front Cell Neurosci. 2021 Sep 13;15:661838. doi: 10.3389/fncel.2021.661838 (PMC8475767; doi:10.3389/fncel.2021.661838)
Supplement: Supplementary file 1 [file Table_1.docx]

**Supplementary table 1. MicroRNAs and long non-coding RNAs involved in BBB dysfunction and related cellular events in BMECs**

| Non-coding RNAs | Species | Cellular events in BMECs | Target gene | References |
| --- | --- | --- | --- | --- |
| MicroRNAs |  |  |  |  |
| miR-23a-3p | human | ZO-1 expression ↓ | ZO-1 | Hu et al., 2018 |
| miR-96 | human | miR-96 levels after treatment with granulocyte-macrophage colony-stimulating factor ↑  ZO-1 expression ↓ | Erythroblast transformation-specific (ETS) transcription factor ERG | Zhang et al., 2018 |
| miR-101 | human | miR-101 levels after treatment with HIV-1 Tat C ↑  VE-cadherin ↓  Claudin-5 expression ↓ | VE-cadherin | Mishra and Singh, 2013 |
| miR-143 | human | miR-143 levels after treatment with methamphetamine ↑  Permeability ↑  ZO-1 expression ↓  Occludin expression ↓  Claudin-5 expression ↓ | p53 unregulated modulator of apoptosis | Bai et al., 2016 |
| miR-155 | human | Permeability (Fluorescein-Dextran) ↑ | Claudin-1 | Pena‐Philippides et al., 2018 |
| miR-212/132 | human | miR-212/132 levels under the condition of oxygen-glucose deprivation (OGD) ↑  miR-212/132 levels under the condition of OGD/reoxygenation → | Unknown | Burek et al., 2019 |
|  | mouse | miR-212/132 levels under the condition of OGD ↑  miR-212/132 levels under the condition of OGD/reoxygenation ↑  TEER ↓  Claudin-1 expression ↓  Expression of tight junction-associated protein 1 (Tjap1) ↓ | Claudin-1, Tjap1 | Burek et al., 2019 |
| miR-424-5p | human | miR-424-5p levels after treatment with amyloid-β ↑  Permeability (horseradish peroxidase) ↑  TEER ↓  ZO-1 expression ↓  Occludin expression ↓ | Endophilin-1 | Lin et al., 2019 |
| miR-150 | rat | Permeability (FITC-Dextran) ↑  Claudin-5 expression ↓ | Angiopoietin receptor Tie-2 | Fang et al., 2016 |
| miR-130a | rat | miR-130a levels under the condition of OGD ↑  Permeability (FITC-Dextran) ↑  Occludin expression ↓ | Homeobox A5 | Wang et al., 2018 |
| miR-21-3p | mouse | miR-21-3p levels under the condition of OGD ↑  Permeability (horseradish peroxidase) ↑  ZO-1 expression ↓  Occludin expression ↓  Claudin-5 expression ↓ | Methionine adenosyltransferase 2B | Ge et al., 2019 |
| miR-29b | mouse | miR-29b levels after treatment with homocysteine ↑  Permeability (FITC-albumin) ↑ | (DNMT3b) | Kalani et al., 2014 |
| miR-30a | mouse | miR-30a levels under the condition of OGD ↑  TEER ↓  Occludin expression ↓  Claudin-5 expression ↓ | Zinc transporter ZnT4 | Wang et al., 2021 |
| miR-34a | mouse | Permeability (FITC-Dextran) ↑ | Cytochrome C | Bukeirat et al., 2016 |
| miR-182 | mouse | miR-182 levels under the condition of OGD ↑  ZO-1 expression ↓  Occludin expression ↓  Claudin-5 expression → | Unknown | Zhang et al., 2019 |
| miR-501-3p | mouse | TEER ↓  ZO-1 expression ↓  Occludin expression →  Claudin-5 expression → | ZO-1 | Toyama et al., 2018 |
| Long non-coding RNAs |  |  |  |  |
| LINC00094 | human | LINC00094 levels after treatment with amyloid-β ↑  Permeability (horseradish peroxidase) ↑  TEER ↓  ZO-1 expression ↓  Occludin expression ↓  Claudin-5 expression ↓ | miR-224-5p, miR-497-5p | Zhu et al., 2019 |
| LINC00662 | human | LINC00662 levels after treatment with amyloid-β ↑  Permeability (horseradish peroxidase) ↑  TEER ↓  ZO-1 expression ↓  Occludin expression ↓  Claudin-5 expression ↓ | ETS-domain protein 4 (ELK4) | Liu et al., 2020 |
| Small nucleolar RNA host gene 3 (Snhg3) | rat | Snhg3 levels after treatment with OGD plus hemin ↑  Permeability (FITC-Dextran) ↑ | Unknown | Zhang et al., 2019 |
| LOC102640519 | mouse | LOC102640519 levels after treatment with OGD/R plus VEGF ↑  Permeability (horseradish peroxidase) ↑  TEER ↓  ZO-1 expression ↓  Occludin expression ↓  Claudin-5 expression ↓ | HOXC13 | Wu et al., 2018 |
